# Simultaneous untargeted and targeted metabolomics profiling of underivatized primary metabolites in sulfur-deficient barley by ultra-high performance liquid chromatography-quadrupole/time-of-flight mass spectrometry

**DOI:** 10.1186/s13007-018-0329-0

**Published:** 2018-07-24

**Authors:** Hikmat Ghosson, Adrián Schwarzenberg, Frank Jamois, Jean-Claude Yvin

**Affiliations:** 1 Centre Mondial de l’Innovation Roullier (CMI), 18 Avenue Franklin Roosevelt, 35400 Saint-Malo, France; 2 UFR Sciences et Propriétés de la Matière, Université de Rennes 1, 2 rue du Thabor, CS 46510, 35065 Rennes Cedex, France

**Keywords:** Plant metabolomics, Primary metabolites, Organic acids, Amino acids, LC–HRMS, Sulfur deficiency

## Abstract

**Background:**

Metabolomics based on mass spectrometry analysis are increasingly applied in diverse scientific domains, notably agronomy and plant biology, in order to understand plants’ behaviors under different stress conditions. In fact, these stress conditions are able to disrupt many biosynthetic pathways that include mainly primary metabolites. Profiling and quantifying primary metabolites remain a challenging task because they are poorly retained in reverse phase columns, due to their high polarity and acid–base properties. The aim of this work is to develop a simultaneous untargeted/targeted profiling of amino acids, organic acids, sulfur metabolites, and other several metabolites. This method will be applied on sulfur depleted barley, in order to study this type of stress, which is difficult to detect at early stage. Also, this method aims to explore the impact of this stress on barley’s metabolome.

**Results:**

Ultra-high performance liquid chromatography–high resolution mass spectrometry-based method was successfully applied to real samples allowing to discriminate, detect, and quantify primary metabolites in short-runs without any additional sampling step such as derivatization or ion pairing. The retention of polar metabolites was successfully achieved using modified C18 columns with high reproducibility (relative standard deviation below 10%). The quantification method showed a high sensitivity and robustness. Furthermore, high resolution mass spectrometry detection provided reliable quantification based on exact mass, eliminating potential interferences, and allowing the simultaneous untargeted metabolomics analysis. The untargeted data analysis was conducted using Progenesis QI software, performing alignment, peak picking, normalization and multivariate analysis. The simultaneous analysis provided cumulative information allowing to discriminate between two plant batches. Thus, discriminant biomarkers were identified and validated. Simultaneously, quantification confirmed coherently the relative abundance of these biomarkers.

**Conclusions:**

A fast and innovated simultaneous untargeted/targeted method has successfully been developed and applied to sulfur deficiency on barley. This work opens interesting perspectives in both fundamental and applied research. Biomarker discovery give precious indication to understand plant behavior during a nutritional deficiency. Thus, direct or indirect measurement of these compounds allows a real time fertilization management and encounter the challenges of sustainable agriculture.

**Electronic supplementary material:**

The online version of this article (10.1186/s13007-018-0329-0) contains supplementary material, which is available to authorized users.

## Background

Metabolomics based on gas chromatography–mass spectrometry (GC–MS) and/or liquid chromatography–mass spectrometry (LC–MS) are widely applied for exhaustive and specified studies in many different scientific fields [1]. Both untargeted and targeted strategies are being developed, notably in agronomy and plant biology [2].

Many different metabolomics studies in plant biology are emerging for various aims (e.g. discovering new biocontrol agents, phytomedicine, etc.). Otherwise, in order to understand plant physiological reactions and behaviors under different biotic and/or abiotic stress (e.g. drought stress, nutrient deficiencies, bio-stimulant applications, microorganism effects, associated crop), high-throughput methods need to be developed. This is to identify and/or quantify involved biomarkers in a complex matrix. The different types of stress or treatments are able to modify and disrupt biosynthesis pathways [3–8]. Furthermore, biomolecules implied in these pathways, mainly primary and polar metabolites, are present in low concentrations, leading to many difficulties during the extraction and chromatographic separation. The high polarity and acid–base properties implies time consuming sample preparation (e.g. derivatization). Moreover, delicate chromatographic optimization should be achieved, in order to assure reliable and robust separation and detection with GC–MS and/or LC–MS systems.

Several targeted methods based on LC–MS have been reported in the literature [4, 9–22], mainly for primary metabolites analysis [9–22]. However, most of LC–MS methods needed additional sampling steps [15, 18–20], or were performed using hydrophilic interaction liquid chromatography (HILIC) [4, 12, 21, 22]. HILIC methods required a delicate, careful optimization, and relatively longtime column conditioning. On the other hand, ion pairing technique was also applied [9, 16, 17, 22], however it risks the residual system contamination. Liu et al. [13] reported a targeted high performance liquid chromatography–mass spectrometry (HPLC–MS) method that allowed to analyze 28 polar metabolites in 25 min without additional sampling steps.

Alternatively, several untargeted GC–MS based methods applied in plants have been reported [2, 3, 6, 23–26]. GC–MS based techniques are mainly utilized for primary metabolites profiling [23, 27], providing high chromatographic resolution and high sensitivity. Moreover, the high reproducibility of electronic impact (EI) fragmentation provides reliable metabolites identification [27]. Nevertheless, samples required derivatization to analyze these non-volatile and polar metabolites. Furthermore, thermolabile metabolites analysis are difficult due to thermo-degradation [27].

Hence, untargeted LC–MS based methods are rapidly developing lately [28]. Due to their performance to analyze wide variety of metabolites [27], many LC–MS based methods were recently developed and increasingly applied for metabolomics profiling in plants [23, 29–33] and phytomedicine [34, 35].

It is worth to mention that simultaneous targeted and untargeted metabolomics was applied in a phytomedicine study [36]. However, none of the reported studies showed this approach on the study of primary metabolites in plant metabolomics. Our objective was to develop, validate and apply an ultra-high performance liquid chromatography–high resolution mass spectrometry (UPLC–HRMS) based metabolomics method for analyzing underivatized primary metabolites in less than 10 min.

As primary metabolites play an essential role in plant growth, development, and reproduction, and secondary metabolites as flavonoids and polyphenols are involved in plant defense [37, 38], their abundances explain an important part of plant behaviors under different biotic or abiotic stress [3–8, 27]. Consequently, 34 major metabolites were selected from different biochemical pathways in order to explain the physiological behavior under nutrient deprival; sulfur-deficiency in our case.

In fact, sulfur deficiency can lead to yield losses due to its non-visual symptoms, it is not easily identifiable because of the confusion between sulfur deficiency and nitrogen deficiency [39]. Moreover, early stage sulfur deficiency is also difficult to be detected, due to the usual less accurate prediction when the sampling is on the early growth stage. However, analyzing biochemical indicators as glutathione can lead to more reliable diagnosis [40].

**Fig. 1:**
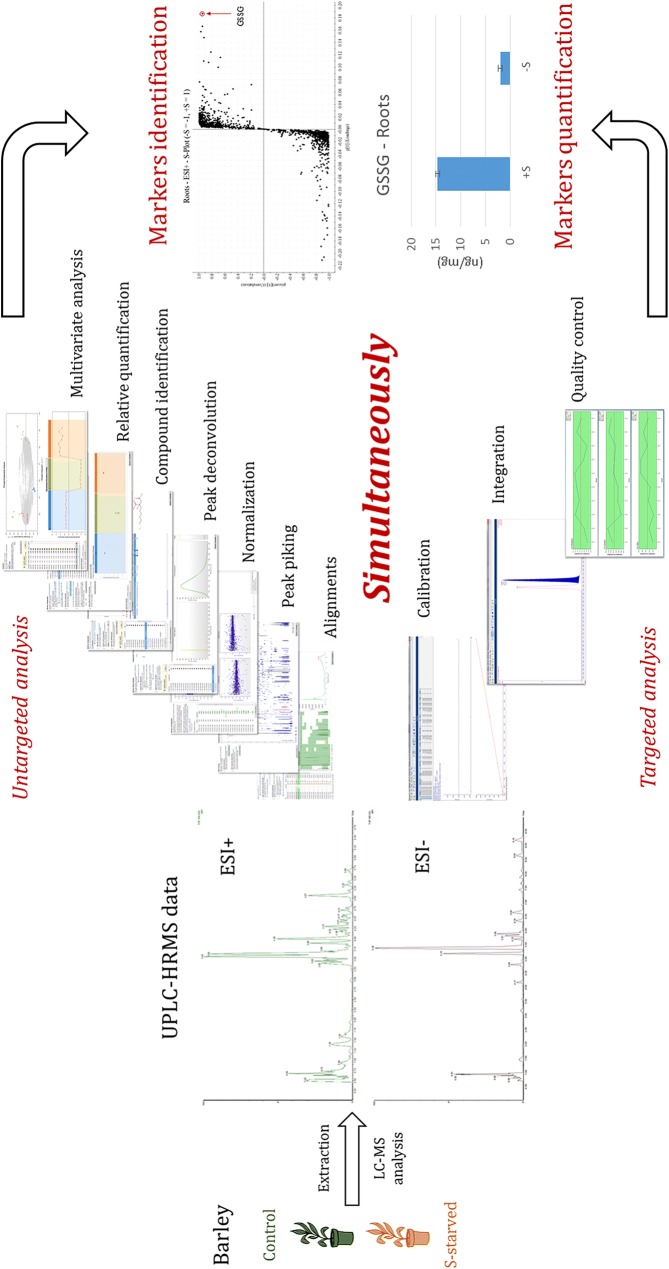
Untargeted/targeted UPLC–HRMS^e^ workflow

The workflow represented in Fig. 1 consists of extracting metabolites from plant material, realizing LC–HRMS analysis, and processing same data with two different approach: untargeted profiling for batch discrimination and biomarkers determination, and targeted quantification and biomarkers identification.

## Methods

### Chemicals and reagents

Amino acids, and organic acids HPLC quality standards proline (Pro), isoleucine (Ile), leucine (Leu), asparagine (Asn), aspartic acid (Asp), glutamine (Gln), glutamic acid (Glu), lysine (Lys), methionine (Met), histidine (His), phenylalanine (Phe), arginine (Arg), tyrosine (Tyr), tryptophan (Trp), 5 amino acid derivatives: O-acetyl-serine, thiamine, glutathione reduced (GSH), S-adenosyl-methionine (SAM), glutathione oxidized (GSSG), fumaric acid, succinic acid, malic acid, phospho(enol)pyruvic acid, cis-aconitic acid, shikimic acid, citric acid, isocitric acid, gluconic acid, 2 phosphorylated sugars: d-glucose 6-phosphate, trehalose 6-Phosphate, 4 secondary metabolites: gallic acid, azelaic acid, kaempferol, chlorogenic acid, and two internal standards: Lactitol and Taurine, were purchased from Sigma-Aldrich (Saint Quentin, France).

Ultra-pure water was prepared by a Milli-Q Advantage A10 system (Darmstadt, Germany), Acetonitrile (ACN) and Methanol (MeOH) Optima LC–MS grade were purchased from Fisher (Leicester, UK), Formic Acid (FA) LC–MS grade and perchloric acid (PCA) were purchased from Merck (Darmstadt, Germany).

### Plant materials

Method was applied on barley (*Hordeum vulgare*) plants grown and treated in Centre Mondial de l’Innovation Roullier greenhouse (Saint-Malo, Bretagne, France). Seeds of *Hordeum vulgare* cv. Irina were germinated on vermiculate for 3 days in the dark and for additional 4 days under light conditions. After 1 week, seedlings were transplanted to a 5.9 L tank in greenhouse that was set to a 14/10 h day/night cycle at a day/night temperature of 28/25 °C with 40–50% relative humidity. Plants were divided into two batches: (1) S-sufficient (0.5 mM) plants were grown with a complete nutritive solution, (2) S-deficient plants were grown in a different solution with a low concentration of S (0 mM): Ca(NO_3_)^2−^ 2 mM, K_2_HPO_4_ 1 mM, MgCl_2_ 0.5 mM, NH_4_H_2_PO_4_ 0.5 mM, CaCl_2_ 0.5 mM, H_3_BO_3_ 0.001 mM, MnNO_3_ 0.0025 mM, ZnNO_3_ 0.0005 mM, CuNO_3_ 0.0002 mM, (NH_4_)_6_Mo_7_O_24_ 0.00001 mM and EDTA, 2NaFe 0.1 mM. The nutrient solution was buffered to pH 5.9 and renewed every 2 days and continuously aerated. After 2 weeks of stress, leaves and roots of two batches were harvested and immediately frozen in liquid nitrogen and then stored at − 80 °C until analysis. Before extraction, materials were grinded in CryoMill (5 µm, Retsch, Haan, Germany).

### UPLC conditions

Ultra-high performance liquid chromatography analysis was performed using a Waters Acquity H-Class UPLC system (Waters Corp, Milford, USA). Amino acids and sulfur contain metabolites separation was performed using a Waters UPLC HSS T3 column (2.1 × 100 mm, 1.8 µm). The mobile phase consisting of water containing 0.1% formic acid (A) and acetonitrile/methanol 50:50 v/v containing 0.1% formic acid (B) was applied with the optimized gradient elution as follows: 100% A at 0–1.5 min, 100–80% A at 1.5–2 min, 80–20% A at 2–2.5 min, 20% A at 2.5–4.5 min, 20–100% A at 4.5–5 min, 100% A at 5–7 min. The flow rate was kept at 0.4 mL/min, and column temperature was maintained at 25 °C.

The separation of organic acids, phosphorylated sugars, secondary metabolites and two amino acids (aspartic acid and glutamic acid) was achieved using a Phenomenex Luna^®^ Omega PS C18 (100 × 2.1 µm, 1.6 µm) column (Torrance, USA). The mobile phase consisting of water containing 0.5% formic acid (A) and methanol/water 70:30 v/v containing 0.5% formic acid (B) was applied with the optimized gradient elution as follows: 100% A at 0–1 min, 100–20% A at 1–4 min, 20–0% A at 4–6.5 min, 0% A at 6.5–7.5 min, 0–100% A at 7.5–7.9 min, 100% A at 7.9–10 min. The flow rate was kept at 0.3 mL/min, column temperature was maintained at 35 °C. The injection volume for both columns was 10 µL and samples were maintained at 10 °C.

### QToF conditions

High resolution mass spectrometry detection of metabolites was performed by Waters Xevo G2-S quadrupole/time-of-flight mass spectrometer (QToF MS) (Waters Corp, Milford, USA) equipped with an electrospray ionization (ESI) source. For positive ESI, source voltage was set to 0.5 kV and cone voltage was 15 V, whereas source temperature was maintained at 130 °C with a cone gas flow of 20 L/h. Desolvation temperature was at 500 °C with desolvation gas flow of 800 L/h. For negative ESI, source voltage was set to 2.5 kV and cone voltage was 30 V, whilst source temperature was maintained at 130 °C with a cone gas flow of 20 L/h, desolvation temperature was at 550 °C with desolvation gas flow of 900 L/h. Leucine-Enkephalin (Waters, Manchester, UK) was used as lockmass reference, (ion at *m/z* 556.2771 in positive mode and *m/z* 554.2615 in negative mode), which was introduced by a Lockspray at 10 μL/min for real-time data calibration. The MS^E^ data were acquired in centroid mode using a scan range 50–800 Da, scan time 0.1 s, resolution was set at 20000 full width half maximum (FWHM), and a collision energy ramp 40–80 V.

Molecular ions [M + H]^+^ and [M − H]^−^ were detected in positive and negative ionization, respectively. Chromatographic peaks were extracted from the full scan chromatograms using MassLynx V4.1 software (Waters Inc., USA), based on [M + H]^+^ and [M − H]^−^ ions. Peak areas were integrated using TargetLynx software (Waters Inc., USA), and data treatment for untargeted analysis were performed by Progenesis QI software (Nonlinear Dynamics, Newcastle, UK) and EZinfo 3.0 software (Umetrics AB, Umeå, Sweden).

### Method validations

Determination of the limit of detection (LOD), limit of quantitation (LOQ), and linearity were carried out using a series of diluted mixed standards of metabolites. The concentrations were chosen through preliminary tests to establish the linear range and enable quantification in the plant material of interest.

To determine the method precision, three concentration levels (one close to LOQ, one intermediate and one close to the upper limit of linear range) of mixed standards were injected ten times. Otherwise, different plant samples were injected ten times for intra-sample validation, also 4 biological replicate samples were analyzed for intra-day, and inter-day validation within 6 months. The repeatability and reproducibility intra-sample, intra-day and inter-day for each compound were estimated by calculation of the respective relative standard deviation (RSD) values (Additional file 1: Tables S1–S6).

### Extraction method

20 mg of frozen grinded fresh leaves and roots were weighted in a 2 mL Eppendorf tubes, then 500 µL of cold water/methanol 70:30 v/v (− 20 °C) containing 0.4% of perchloric acid (v/v) solvent were added. Samples were shaken with vortex for 10 min. Then, they were centrifuged using an Eppendorf Centrifuge 5427 R (Hamburg, Germany) for 15 min 12,700 RPM at 4 °C. Supernatants were collected and introduced in a new 2 mL Eppendorf tubes. A second extraction was performed adding 500 µL of water + 0.1% perchloric acid (v/v) to leaves and roots, shaken for 5 min with vortex, and centrifuged for 15 min with 12,700 rotation per minute (RPM) on 4 °C. Then supernatants were recuperated in same tubes of the first extraction. Supernatants were mixed and centrifuged for 10 min in order to eliminate suspended particles without introducing contaminants issued from filters. Finally, supernatants were diluted 2 times with water + 0.1% Formic acid (v/v) and introduced in 2 mL LC–MS vials.

Perchloric acid was used to protect metabolites from enzymatic degradation [41], and to avoid sulfur metabolites oxidation and degradation [14, 42, 43] under basic and neutral condition. Formic acid was used for enhancing electrospray ionization.

### Data treatment

LC–HRMS^E^ acquired data were treated in two different paths: untargeted analysis and targeted analysis.

Untargeted data analysis was performed using Progenesis QI software. Data were processed in successive treatment steps as peak alignment, peak picking and normalization to obtain data matrix. This matrix was used to perform multivariate analysis.

Targeted analysis was performed using TargetLynx software. Targeted metabolites’ *m/z* ratios were extracted from chromatogram, and chromatographic peaks were integrated to quantify metabolites using calibration curves with internal standards correction. Chosen internal standards were Taurine for positive ionization mode and Lactitol for negative ionization mode. [M + H]^+^ and [M − H]^−^ ions were used for quantification.

### Method application

The method was evaluated on barley under sulfur controlled and deficient conditions. Leaves and roots were analyzed separately for untargeted/targeted metabolite profiling. The data issued from the LC–HRMS analysis were used to perform the untargeted data analysis and targeted quantification (Fig. 1). Quality control solution (QC) was prepared by mixing similar volume aliquots from all samples. QC solution was prepared in order to obtain the variability of all samples.

Analytical sequence consisted in a calibration curve followed by 10 consecutive injections of QC to stabilize the LC system. Then, all samples were injected randomly to minimize the effect of instrumental drift. A QC was injected every 5 samples as well as a standard QC in order to control carry over, stability and robustness.

## Results and discussion

### Optimization and validation of targeted profiling method

#### Chromatographic separation

**Fig. 2:**
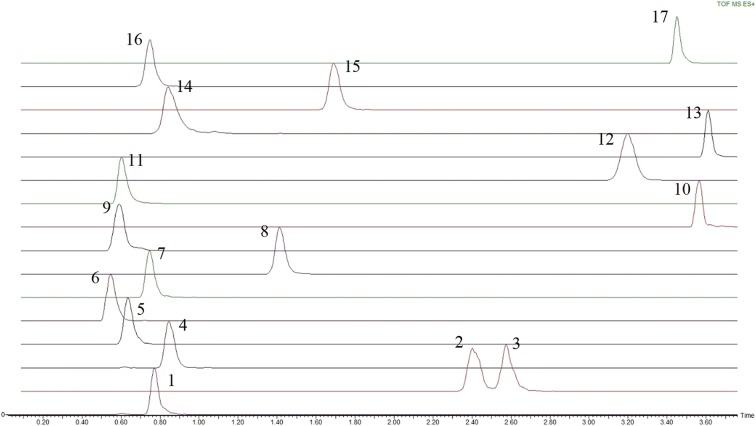
Extracted ion chromatogram of amino acids and sulfur metabolites—waters acquity UPLC HSS T3 column—ESI+: 1, Proline; 2, Isoleucine; 3, Leucine; 4, Asparagine; 5, Glutamine; 6, Lysine; 7, O-Acetyl-serine; 8, Methionine; 9, Histidine; 10, Phenylalanine; 11, Arginine; 12, Tyrosine; 13, Tryptophan; 14, Thiamine; 15, Glutathione reduced; 16, S-adenosyl-methionine; 17, Glutathione oxidized

Amino acids and sulfur containing metabolites separation was performed using an HSS T3 column. The majority of compounds (13 out of the 17 compounds) were chromatographically separated (Fig. 2). Structural isomers Isoleucine and Leucine are chromatographically resolved as shown in Fig. 4. Co-eluting compounds as Histidine and Arginine can be differentiated by their mass difference. The HSS T3 column contains a modified C18 stationary phase with 100% silica base, which provides a hydrophilic interaction with polar metabolites, allowing to enhance their retention. Furthermore, this 100% silica base allows to use 100% aqueous eluting phase, so polar metabolites are weakly eluting.

**Fig. 3:**
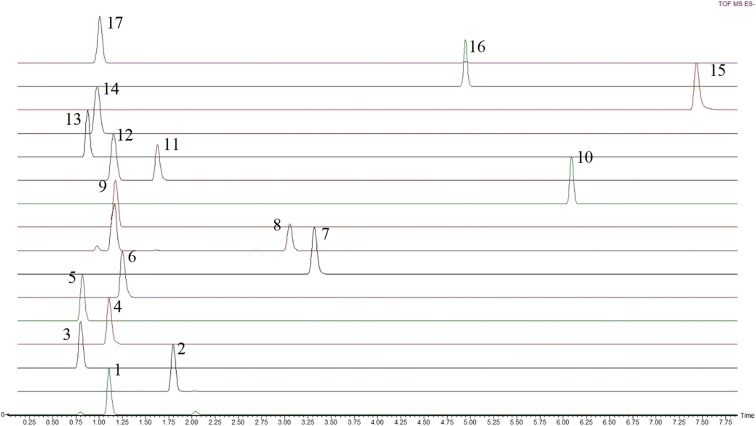
Extracted ion chromatogram of organic acids—Phenomenex Luna^®^ Omega PS C18 column—ESI: 1, Fumaric acid; 2, Succinic acid; 3, Aspartic acid; 4, Malic acid; 5, Glutamic acid; 6, Phospho(enol)pyruvic acid; 7, Gallic acid; 8, Cis-Aconitic acid; 9, Shikimic acid; 10, Azelaic acid; 11, Citric acid; 12, Isocitric acid; 13, Gluconic acid; 14, d-Glucose 6-Phosphate/Fructose-6-Phosphate; 15, Kaempferol; 16, Chlorogenic acid; 17, Trehalose 6-Phosphate

**Fig. 4:**
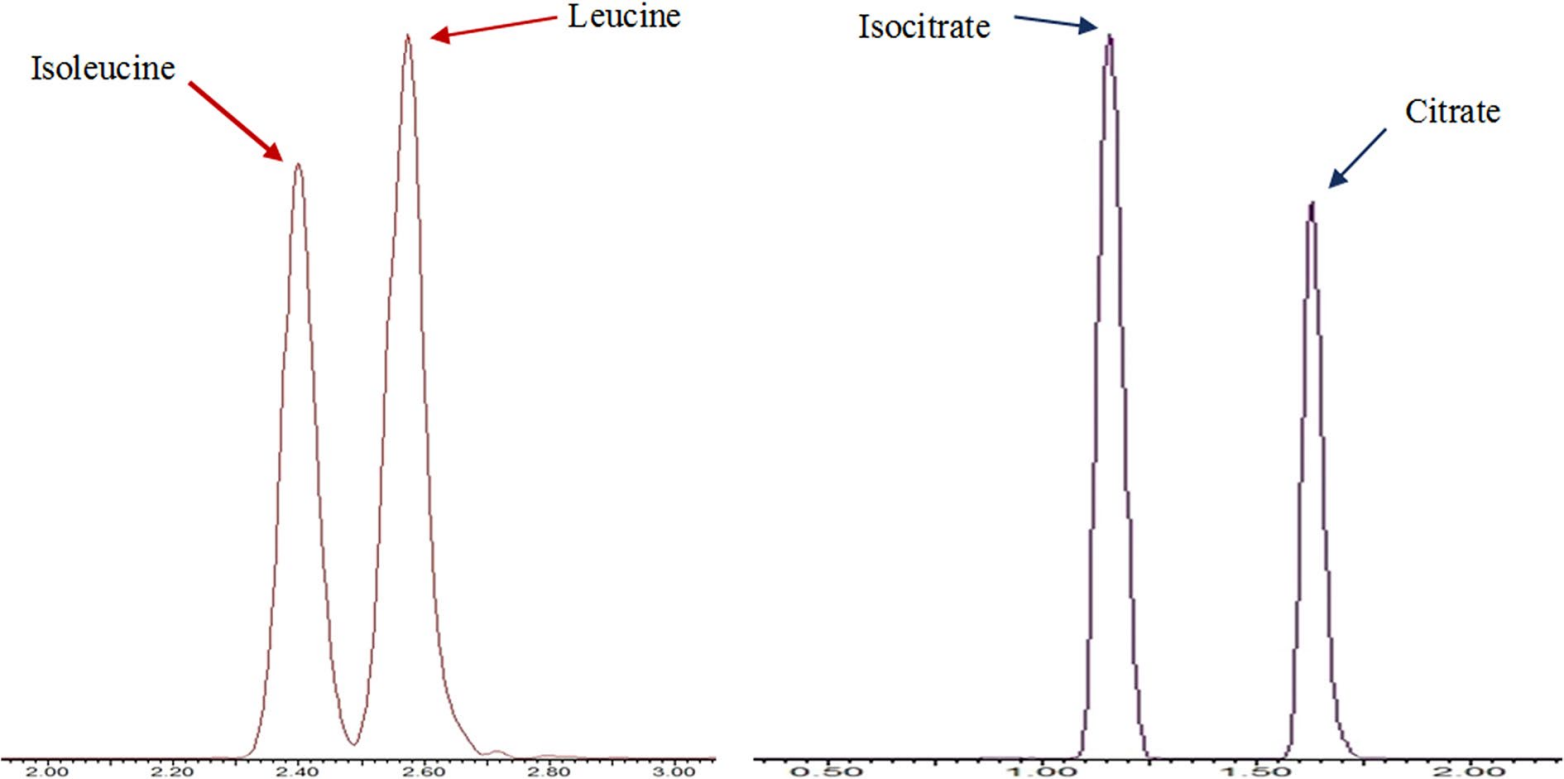
Separated isomers, amino acids isoleucine and leucine using an HSS T3 column and organic acids isocitrate and citrate using a Luna Omega PS column

Organic acids and other metabolites including phosphorylated sugars, secondary metabolites and two amino acids aspartate and glutamate were separated using a Luna^®^ Omega PS C18 column that allowed to chromatographically resolve 13 of 17 compounds as shown in Fig. 3. The structural isomers isocitrate and citrate were completely separated as represented in Fig. 4, whereas co-eluting analytes as fumarate and malate can be differentiated by their mass difference. However, the phosphorylated sugar d-glucose 6-phosphate was detected but it could not be separated from its isomer d-fructose 6-phosphate. The Luna^®^ Omega PS C18 column is also a modified C18 stationary phase, including positive charge implanting. This positive charge allows strong retention of organic acids, due to the charge interaction with carboxyl function. Additionally, the 100% aqueous eluting phase is applicable with this column.

Detection and quantification were bolstered using high resolution QToF mass spectrometer, which is able to discriminate between metabolites’ ions with the same nominal mass (e.g. cis-aconitate and shikimate). High resolution detection also allowed the elimination of potential interferences as isotope contributions. In fact, reliability of quantification by high resolution mass spectrometry is assured by exact mass, due to the elimination of potential errors issued from interferences [44]. Furthermore, HRMS acquisition is necessary to apply untargeted analysis of acquired data.

For cis-aconitate, two chromatographic peaks corresponding to its accurate mass (*m/*z 173.0092) were reported. One of the two peaks corresponds to the same retention time of that of Isocitrate. This peak represents a fragment issued from isocitrate giving an ion with the same elemental composition that the cis-aconitate (water loss) (Additional file 1: Figures S1 and S2).

We have found that amino acids can be detected in both positive mode (as protonated ions) and negative mode (as deprotonated ions). Most of amino acids have shown a better response in positive mode. However, aspartic acid and glutamic acid showed a better signal in negative mode. On the other hand, O-acetyl-serine, methionine, tryptophan, glutathione reduced and glutathione oxidized were more sensitive in positive mode. The introduction of primary metabolites with an *m/z* below 100 (Pyruvate as an example) was difficult due to instrumental limits.

#### Limit of detection, limit of quantification and linearity

The LOQ was determined as the smallest amount of a compound reliably quantified showing a signal to noise (S/N) value above 10, and the LOD value is the smallest amount of a compound that can be reliably distinguished, usually showing an S/N value above 3. The linear range was determined using 4 replicates of successively diluted mix of standards.

Positive ionization mode showed a very sensitive detection for amino acid and sulfur metabolites. Calculated LOQ was 10 ng mL^−1^ and lower for all amino acids and sulfur metabolites, as shown in Table 1. Only asparagine showed a LOQ higher than 10 ng mL^−1^.

**Table 1:** Retention times (RT), linearity ranges, LOD and LOQ of polar metabolites tested

Compound	Elemental composition	Ions detected	Measured *m/z*	RT (min)	Linearity range (ng mL^−1^)	R^2^	LOD (ng mL^−1^)	LOQ (ng mL^−1^)
*ESI*−								
Fumaric acid	C_4_H_4_O_4_	[M − H]^−^	115.0037	1.10	200–10,000	0.9995	75	200
Succinic acid	C_4_H_6_O_4_	[M − H]^−^	117.0193	1.80	500–10,000	0.9996	150	500
Aspartic acid	C_4_H_7_NO_4_	[M − H]^−^	132.0302	0.80	250–5000	0.9966	15	50
Malic acid	C_4_H_6_O_5_	[M − H]^−^	133.0142	1.10	100–5000	0.9975	15	50
Glutamic acid	C_5_H_9_NO_4_	[M − H]^−^	146.0459	0.82	50–2500	0.9993	15	50
Phospho(enol)pyruvic acid	C_3_H_5_O_6_P	[M − H]^−^	166.9751	1.25	1000–10,000	0.9999	200	500
Gallic acid	C_7_H_6_O_5_	[M − H]^−^	169.0142	3.32	100–5000	0.9997	0.75	2.5
Cis-aconitic acid	C_6_H_6_O_6_	[M − H]^−^	173.0092	3.06	250–5000	0.9969	75	250
Shikimic acid	C_7_H_10_O_5_	[M − H]^−^	173.0455	1.18	100–2500	0.9998	4.5	15
Azelaic acid	C_9_H_16_O_4_	[M − H]^−^	187.0976	6.09	10–1000	0.9998	0.5	1.5
Citric acid	C_6_H_8_O_7_	[M − H]^−^	191.0197	1.63	50–1000	0.9969	5	20
Isocitric acid	C_6_H_8_O_7_	[M − H]^−^	191.0197	1.16	50–1000	0.9983	5	20
Gluconic acid	C_6_H_12_O_7_	[M − H]^−^	195.0510	0.88	50–1000	0.9968	4	15
Kaempferol	C_15_H_10_O_6_	[M − H]^−^	285.0405	7.44	20–1000	0.9951	0.5	2
Chlorogenic acid	C_16_H_18_O_9_	[M − H]^−^	353.0878	4.94	20–1000	0.9978	1.5	5
Trehalose 6-Phosphate	C_12_H_23_O_14_P	[M − H]^−^	421.0753	1.01	50–1000	0.9955	0.5	1.5
*ESI*+								
Proline	C_5_H_9_NO_2_	[M + H]^+^	116.0706	0.77	2.5–125	0.9981	0.75	2.5
Isoleucine	C_6_H_13_NO_2_	[M + H]^+^	132.1019	2.41	6.5–125	0.9984	2	6.5
Leucine	C_6_H_13_NO_2_	[M + H]^+^	132.1019	2.58	6.5–125	0.9994	2	6.5
Asparagine	C_4_H_8_N_2_O_3_	[M + H]^+^	133.0608	0.85	20–100	0.9972	6	20
Glutamine	C_5_H_10_N_2_O_3_	[M + H]^+^	147.0764	0.64	10–50	0.9918	0.3	1
Lysine	C_6_H_14_N_2_O_2_	[M + H]^+^	147.1128	0.54	7.5–150	0.9987	2	7.5
O-Acetyl-serine	C_5_H_9_NO_4_	[M + H]^+^	148.0604	0.74	10–100	0.9964	5	10
Methionine	C_5_H_11_NO_2_S	[M + H]^+^	150.0583	1.41	15–150	0.9981	0.5	1.5
Histidine	C_6_H_9_N_3_O_2_	[M + H]^+^	156.0768	0.60	8–150	0.9982	0.5	1.5
Phenylalanine	C_9_H_11_NO_2_	[M + H]^+^	166.0863	3.57	3–200	0.9990	0.15	0.5
Arginine	C_6_H_14_N_4_O_2_	[M + H]^+^	175.1190	0.60	9–200	0.9968	0.05	0.2
Tyrosine	C_9_H_11_NO_3_	[M + H]^+^	182.0812	3.20	9–200	0.9986	0.5	2
Tryptophan	C_11_H_12_N_2_O_2_	[M + H]^+^	205.0972	3.61	2–100	0.9989	0.35	1.2
Thiamine	C_12_H_17_ClN_4_OS	[M + H]^+^	265.1118	0.83	2–100	0.9999	0.5	2
Glutathione reduced	C_10_H_17_N_3_O_6_S	[M + H]^+^	308.0911	1.69	5–100	0.9996	0.15	0.5
S-adenosyl-methionine	C_15_H_22_N_6_O_5_S	[M + H]^+^	399.1445	0.74	10–100	0.9974	0.75	2.5
Glutathione oxidized	C_20_H_32_N_6_O_12_S_2_	[M + H]^+^	613.1592	3.45	10–100	0.9974	0.5	1

For ESI−, organic acids, amino acids, phosphorylated sugars and secondary metabolites showed a LOQ between 1.5 and 500 ng mL^−1^. The negative ionization was sensitive enough to detect and quantify all organic acids in plant samples.

#### Precision

This method showed good reproducibility in retention time and peak area for all standard amino acids and sulfur metabolites detected in positive ionization mode at all injection levels. The overall RSD of ten injections is below 2% for retention time and below 8% for peak area (Additional file 1: Tables S1, S2 and S3).

The precision in retention time and peak area for standard organic acids, amino acids, phosphorylated sugars and secondary metabolites detected in negative ionization was better than that of amino acids and sulfur metabolites. The RSD of ten injections is below 0.7% for retention time and below 8% for peak area at lowest injection level, smaller RSD values for peak area are obtained with intermediate and high injection levels (Additional file 1: Tables S4, S5 and S6).

After targeted optimization and validation, the method was applied for simultaneous targeted and untargeted metabolites profiling on real sample.

### Simultaneous untargeted profiling and quantification of discriminant features

The aim of this application was to discriminate between two batches of barley, one batch under controlled sulfur conditions (+S) and a second batch under sulfur deprivation conditions (−S). Studied samples consisted in 8 biological replicates from each batch, leaves and roots were separately analyzed by two columns: the HSS T3 column in positive polarity and Luna^®^ Omega PS C18 column in negative polarity.

Principal Components Analysis (PCA) is a descriptive unsupervised discrimination analysis that allows to explain variations between different runs without any a priori knowledge of metabolite profiles. After unsupervised analysis, potential features of two groups were exploited using explicative supervised Orthogonal Projections to Latent Structures Discriminant Analysis (OPLS-DA). Features determination was followed by their identification using injected standard solutions. Also, their relative estimated abundance was compared to targeted quantification when they are included in targeted compounds.

Multivariate analysis was performed using Progenesis QI software. Raw LC–HRMS data generated by the instrument were imported to software without any conversion to perform data analysis. A 2D ion intensity map was generated with the retention time and *m/z* information as the ordinate and abscissa respectively. Peak alignment was carried out using a QC run as reference. Alignment score values for all runs were higher than 90%. Peak picking threshold of sensitivity was set at 3, and normalization was performed using all compounds. Time limits and adducts used for each analysis are represented in Additional file 1: Tables S7 and S8.

**Fig. 5:**
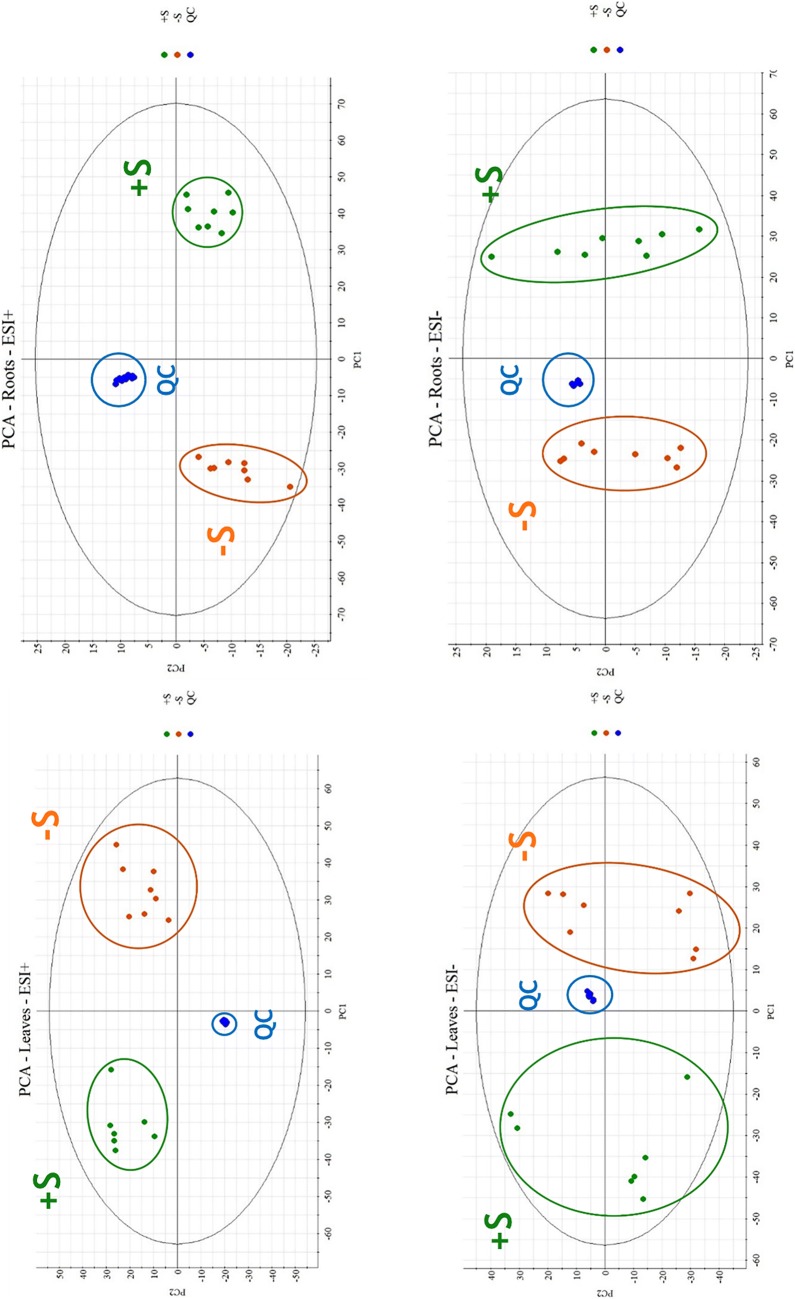
Principal component analysis: upper ESI positive analysis of leaves and roots, lower ESI negative analysis of leaves and roots

Unsupervised PCA was initially applied based on the ions detected in negative and positive modes and filtered by means of a max fold change ≥ 2 and an analysis of variance (ANOVA) *p* value ≤ 0.05 for visualizing the distribution of all the samples. Two-component PCA models accounted of the total variance: 55.14% for leaves and 65.31% for roots in positive ionization, 50.87% for leaves, and 65.94% for roots in negative ionization as shown in Fig. 5.

PCA demonstrated a difference between the two batches, sulfur deficient samples are regrouped (orange) and represented a notable discrimination to control samples (green). Additionally, concentrated grouping of QC runs in blue (Fig. 5), confirms method repeatability demonstrated in method validation.

The two clustered groups were analyzed using a supervised OPLS-DA, in order to find the discriminant features. The S-Plots obtained from OPLS-DA regression allowed to find potential discriminant features as shown in Table 2 and Additional file 1: Figures S5, S6, S7 and S8. Thus, in positive mode, one discriminant molecular feature (3.44_612.1519n) was found in leaves samples, and another (3.47_612.1522n) in roots samples. In negative mode, three discriminant molecular features (0.80_131.0456 *m/z*, 0.81_114.0193 *m/z* and 1.62_191.0190 *m/z*) were found in roots samples. All these features showed the highest variation. Thus, both in-house and online Kegg data-base were used to search and identify these features based on the exact mass, standard retention time (RT), and MS^E^ spectra.

**Table 2:** Identified and putative discriminant molecular features found in leaves and roots samples with untargeted analysis in positive and negative ionization mode

Predicted metabolite	*m/z*	Formula	Mass error (ppm)	Retention time (min)	Anova (*p*)	Pathway	Level of identification confidence^a^
*Leaves*—*ESI*+							
Methyl (1S,2S,7aS)-2-hydroxy-2-methylhexahydro-1H-pyrrolizine-1-carboxylate	217.1543	C_10_H_17_NO_3_	− 0.9	3.64	1.85E−12	Alkaloids derived from ornithine	Level 4
(−)-Swainsonine	156.1021	C_8_H_15_NO_3_	1.1	3.91	6.98E−11	Alkaloids derived from ornithine	Level 4
(2E)-4-(beta-d-Glucopyranosyloxy)-2-(hydroxymethyl)-2-butenenitrile	276.1073	C_11_H_17_NO_7_	− 2.8	3.40	3.73E−09	Cyanogenic glucosides derived from valine or isoleucine	Level 4
Alpha-d-glucopyranosyl-(1->6)-alpha-d-glucopyranosyl-(1->6)-d-glucopyranose	543.1324	C_18_H_32_O_16_	0.5	1.23	5.65E−08		Level 4
Glutathione disulfide	307.0832	C_20_H_32_N_6_O_12_S_2_	− 0.2	3.44	1.55E−05	Sulfur metabolism	Level 1
*Roots*—*ESI*+							
Glutathione disulfide	307.0834	C_20_H_32_N_6_O_12_S_2_	0.4	3.47	7.36E−10	Sulfur metabolism	Level 1
Indoleacrylic acid	188.0710	C_11_H_9_NO_2_	1.0	3.62	8.86E−10	Plant growth hormone	Level 4
p-Coumaric acid	147.0448	C_9_H_8_O_3_	4.7	3.58	4.48E−08	Phenylpropanoid	Level 4
4-(5-Hydroxy-2-methyl-2-azabicyclo[2.2.2]oct-5-yl)-3-methylbutanoic acid	242.1748	C_13_H_23_NO_3_	− 0.9	3.81	0.001502861	Fatty acids	Level 4
*Leaves*—*ESI*−							
Adenosine 5′-monophosphate	346.0546	C_10_H_14_N_5_O_7_P	− 3.4	3.10	2.01E−14	Zeatin biosynthesis	Level 4
2-Hydroxy-3-[6-(methylsulfanyl)hexyl]succinic acid	301.0545	C_11_H_20_O_5_S	4.5	0.80	2.42E−14	Glucosinolates biosynthesis	Level 4
3,5-Dihydroxy-4-oxo-2-phenyl-7-chromanolate	601.1384	C_15_H_11_O_5_^−^	3.9	0.90	5.43E−11	Flavonoid biosynthesis	Level 4
(2S)-2-(Beta-d-glucopyranosyloxy)-3-methyl-3-butenenitrile	304.1028	C_11_H_17_NO_6_	− 4.4	3.57	1.80E−09	Cyanogenic glucosides derived from leucine	Level 4
(2S)-2-(Beta-d-glucopyranosyloxy)-3-methylbutanenitrile	306.1186	C_11_H_19_NO_6_	− 4.4	4.16	8.50E−09	Cyanogenic glucosides derived from leucine	Level 4
Hordatine B-like compounds	625.3092	C_29_H_40_N_8_O_5_	− 4.9	6.19	9.87E−08	Amino acid related compounds	Level 4
(1Z)-3-(2,4-Dihydroxyphenyl)-1-(4-hydroxyphenyl)-3-oxo-1-propen-1-olate	601.1382	C_15_H_11_O_5_^−^	3.6	0.97	3.54E−06	Flavonoid biosynthesis	Level 4
(6aR,11aR)-9-Methoxy-6a,11a-dihydro-6H-[1]benzofuro[3,2-c]chromen-3-yl 6-O-(carboxyacetyl)-beta-d-glucopyranoside	563.1404	C_25_H_26_O_12_	− 0.5	5.43	1.83E−04	Isoflavonoid biosynthesis	Level 4
*Roots*—*ESI*−							
2-Hydroxy-2-[6-(methylsulfanyl)hexyl]succinic acid	301.0547	C_11_H_20_O_5_S	3.4	0.79	1.10E−16	Glucosinolates biosynthesis	Level 4
(4R)-5-Amino-4-hydroxy-2-oxopentanoic acid	128.0347	C_5_H_9_NO_4_	− 4.0	1.58	1.10E−16	Arginine and proline metabolism	Level 4
Dehydroalanine	260.0878	C_3_H_5_NO_2_	− 4.0	0.88	1.11E−16	Cysteine and methionine metabolism	Level 4
dl-Aspartic acid	114.0193	C_4_H_7_NO_4_	− 2.4	0.81	1.11E−16	Amino acids	Level 1
l-(+)-Asparagine	131.0456	C_4_H_10_N_2_O_4_	− 4.1	0.80	4.22E−15	Amino acids	Level 1
5-Hydroxy-2-(3-hydroxy-4-methoxyphenyl)-4-oxo-7-chromanolate	601.1384	C_16_H_13_O_6_^−^	3.6	0.90	5.22E−15	Flavonones biosynthesis	Level 4
Benzoyl-beta-d-glucoside	329.0866	C_13_H_16_O_7_	− 4.4	4.66	2.44E−14	Glycosyl ester	Level 4
Citric acid	191.0190	C_6_H_10_O_8_	− 3.3	1.62	5.17E−13	TCA cycle	Level 1
l-Glutamine	145.0612	C_5_H_10_N_2_O_3_	− 4.2	0.83	5.78E−12	Biosynthesis of amino acids	Level 1
Sinapic acid	245.0423	C_11_H_12_O_5_	− 3.7	0.98	6.99E−12	Phenylpropanoids	Level 4
Oxidized glutathione	611.1441	C_20_H_32_N_6_O_12_S_2_	− 1.1	2.05	8.69E−09	Sulfur metabolism	Level 1

These molecular features were considered as discriminant using OPLS-DA. Glutathione disulfide (GSSG) was identified in positive ionization with [M + 2H]^2+^ ion

^a^According to Schymanski et al. [45]

**Fig. 6:**
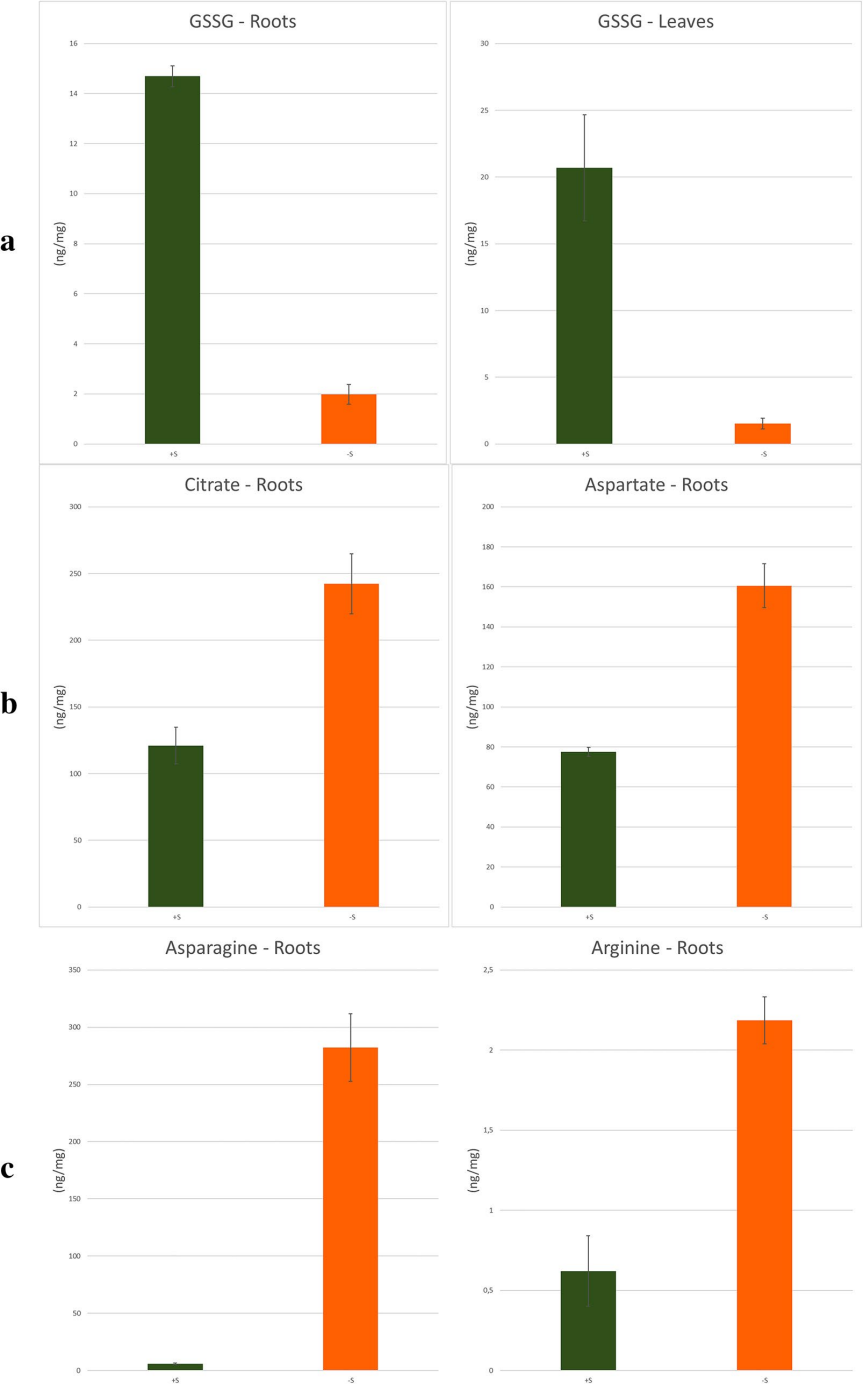
Targeted quantification of identified biomarkers. **a** GSSG in roots and leaves. **b** Citrate and aspartate in roots. **c** Asparagine and arginine in roots

The two discriminant positive mode molecular features (3.44_612.1519n and 3.47_612.1522n) found in leaves and roots samples respectively, were identified as the sulfur metabolite glutathione oxidized (GSSG; see S-Plots in the Additional file 1: Figures S5 and S6). Identification was performed using exact mass, the RT of the standard reference and the MS/MS profile (level 1 of identification confidence [45]). Relative abundance obtained from Progenesis QI showed a low concentration of GSSG in the sulfur stressed group for both leaves and roots (Additional file 1: Figures S9 and S10). This metabolite was quantified using the calibration curve. Targeted quantification results represented in Fig. 6a revealed a coherence with relative quantification. GSSG concentration was notably lower in sulfur stressed plants, which was well explained by the sulfur deficiency in the literature [7, 8]. In fact, glutathione is a regulator of sulfur-uptake and assimilation. Hence, when the plant is sulfur-starved, the decrease of this compound increase transporter activity and maximize sulfate uptake [46].

In silico fragmentations from MS^E^ acquisition of GSSG were also additional information for identification (Additional file 1: Figure S11).

Two metabolites were determined at level 1 in negative mode with OPLS-DA in roots, corresponding to the citric acid as 1.62_191.0190 *m/z* and aspartic acid as 0.81_114.0193 *m/z* (Additional file 1: Figure S7). Their relative abundance was correlated with the targeted quantification as shown in Fig. 6b and Additional file 1: Figures S12 and S13. As found in the quantification, the 2–3 fold of aspartic acid increasing was mentioned by Zhao et al. [40] as one of sulfur deficiency signs.

On the other hand, asparagine was detected and identified at level 1 as 0,80_131,0456 *m/z* in negative mode in roots (Additional file 1: Figure S7) showing a high concentration in sulfur depleted plants while it showed a very low concentration in sulfur sufficient plants (Additional file 1: Figure S14). In fact, asparagine and arginine act as primary and secondary storage of nitrogen respectively in sulfur-depleted plants, as demonstrated by Mertz et al. [47]. Thus, targeted quantification of asparagine and arginine in ESI+ is shown in Fig. 6c, demonstrating a clear coherence with untargeted analysis of asparagine and biological explanation.

**Fig. 7:**
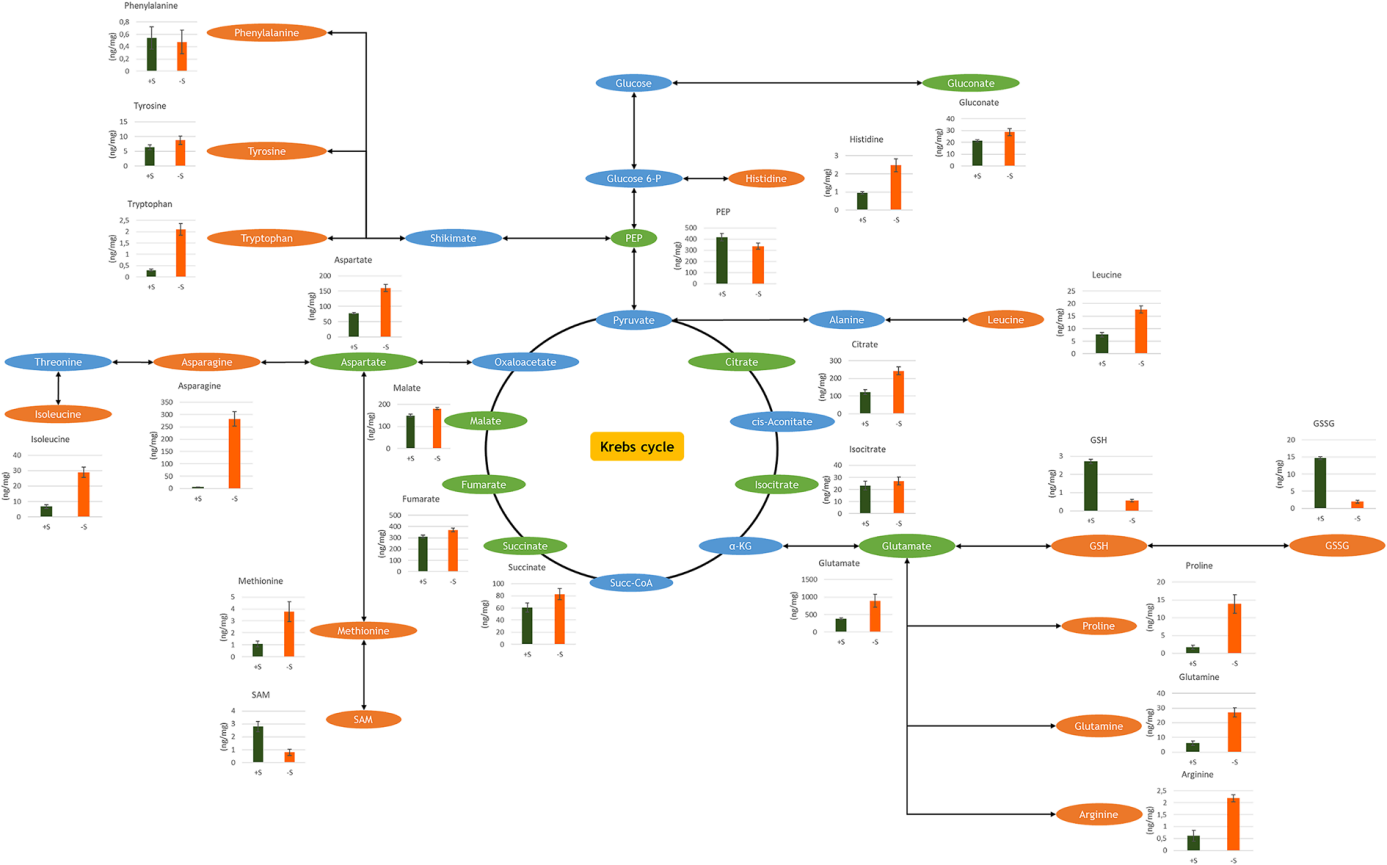
A schematic Krebs cycle pathway based on 23 targeted metabolites quantified in roots using both methods

Hence, according to Schymanski et al. [45], several discriminant molecular features were identified with the level 1 of identification confidence (Table 2). This is by confirming the structure using comparisons with the RT and the MS and MS/MS spectra of reference standards. Other discriminant molecular features represented in Table 2 were identified with the level 4 of identification confidence, due to lack of standard references. The level 4 was reached by elemental compositions identification using exact mass, isotopic patterns, adducts and *in silico* fragmentations. This identification is provided by the software algorithm (Progenesis QI). On the other hand, 1844 molecular features were found in roots (all in both positive and negative ionization modes) and 1573 molecular features were found in leaves (all in both positive and negative ionization modes) after application of the 0.05 *p* value and the ≥ 2 max fold change filters. 272 molecular features could be identified in roots and 342 molecular features in leaves using a barley-specified in-house database. These metabolites were also identified with the level 4 of identification confidence. Otherwise, targeted metabolites quantification in roots is represented in Fig. 7.

### Validation in barley samples

To assess the targeted method of polar metabolite analysis, barley root samples (control batch) were analyzed with both methods. Thus, this method showed a good precision in retention time (RSD below 2%) for ten repeated injections for all detected amino acids and sulfur metabolites (Table 3). All 17 metabolites could be quantified with an intra-day RSD below 8% in comparison between 4 biological replicates (n = 4) and an inter-day RSD (n = 4) below 9% within 6 months (Table 3).

**Table 3:** Intra-sample, intra-day and inter-day validation

Compound	Intra-sample RSD (%) of RT	Intra-day RSD (%) of PA (n = 4)	Inter-day RSD (%) of PA (n = 4)
*ESI*−			
Fumaric acid	0.45	4.70	7.59
Succinic acid	0.34	5.61	8.77
Aspartic acid	0.00	2.42	5.46
Malic acid	0.45	6.97	9.83
Glutamic acid	0.00	5.41	7.07
Phospho(enol)pyruvic acid	2.48	4.88	9.97
Gallic acid	0.27	4.53	N.Q.
Cis-Aconitic acid	0.25	3.94	N.Q.
Shikimic acid	0.62	1.63	N.Q.
Azelaic acid	0.14	5.43	N.Q.
Citric acid	0.28	2.20	3.79
Isocitric acid	0.00	1.79	5.11
Gluconic acid	0.00	4.05	3.51
Kaempferol	0.07	4.70	N.Q.
Chlorogenic acid	0.17	4.27	N.Q.
Trehalose 6-phosphate	0.00	6.37	5.34
*ESI*+			
Proline	0.42	4.02	1.91
Isoleucine	0.46	0.98	3.82
Leucine	0.20	2.32	7.24
Asparagine	0.00	7.024	7.91
Glutamine	0.00	2.32	4.30
Lysine	0.59	4.82	7.28
O-Acetyl-serine	0.96	2.74	4.90
Methionine	0.35	2.95	8.08
Histidine	0.00	3.32	2.85
Phenylalanine	0.13	2.02	3.81
Arginine	0.00	3.89	3.76
Tyrosine	0.35	1.26	7.14
Tryptophan	0.00	4.85	4.60
Thiamine	0.77	5.82	4.11
Glutathione reduced	0.52	6.09	8.94
S-adenosyl-methionine	1.13	5.95	5.78
Glutathione oxidized	0.21	2.48	8.72

Sample RSD (%) of RT was calculated from ten repeated injections of the same extract (replicate 1). Intra-day and inter-day RSD (%) were calculated from four biological replicates. Inter-day quantification was realized within 6 months

*RT* Retention time, *PA* peak area, *N.Q.* not quantified

A comparable precision was obtained in negative mode. Retention time (RSD below 2.5%) showed a good precision for ten repeated injections for all detected organic acids, amino acids, phosphorylated sugars and secondary metabolites (Table 3). Intra-day quantification RSD (n = 4) was below 7%, and inter-day quantification RSD (n = 4) was below 10%. However, gallic acid, cis-aconitic acid, shikimic acid, kaempferol and chlorogenic acid were quantified near the LOQ, and were not quantified after 6 months due to a potential degradation in plant samples (Table 3).

Finally, the simultaneous untargeted/targeted method was successfully applied to real samples, demonstrating high reproducibility with a RSD values below 10%.

It is worth to mention that only 20 mg of fresh material were used to detect 33 underivatized primary metabolites with a high sensitivity and fast analysis at high resolution mass detection. Perchloric acid was added to the solvent in order to reduce the risk of degradation of sulfur containing metabolites. Moreover, untargeted analysis allowed to discriminate between sulfur depleted and controlled barley enabling the identification of several discriminant features related to the primary metabolism under stress conditions.

## Conclusions

A simultaneous untargeted/targeted UPLC–HRMS based method has been developed, providing complementary and reliable information within 7–10 min for a single run, allowing high-throughput analysis. Both UPLC HSS T3 and Luna^®^ Omega PS C18 columns improved considerably retention and chromatographic resolution of polar compounds. The optimized chromatographic conditions allowed to separate 33 primary metabolites including isomers (isoleucine and leucine, isocitrate and citrate) without any derivatization or additional complex sampling step, allowing simple, rapid, and reproducible analysis of these metabolites, but also allowing untargeted metabolic profiling. On the other hand, high resolution mass spectrometry provided high selectivity for untargeted analysis. It also provides reliable and sensitive compound detection and quantification with accurate mass measurement in complex samples, which allowed to discriminate between compounds with the same nominal mass, potential co-eluted interferences, and isotopes contributions. The MS^E^ data acquisition supplied a structural information that can be used for compound identification. The method has succeeded to discriminate between different plant batches under sulfur controlled/deficient, and allowed to identify several biomarkers confirmed by the untargeted/targeted profiling analysis. This work opens interesting perspectives in both fundamental and applied research. Indeed, biomarkers give precious indication on the mechanisms that govern the plant nutrition, especially during a nutritional deficiency. The development of decision support tools based on a direct or indirect measurement of these metabolites would be promising for the plant nutritional status, thus allowing a real time fertilization management and encounter the challenges of sustainable agriculture.

## Additional file

**Additional file 1.** **Table S1.** RSD – ESI+ – Lowest concentration. **Table S2.** RSD – ESI+ – Intermediate concentration. **Table S3.** RSD – ESI+ – Highest concentration. **Table S4.** RSD – ESI- – Lowest concentration. **Table S5.** RSD – ESI- – Intermediate concentration. **Table S6.** RSD – ESI- – Highest concentration. **Table S7.** Adducts list. **Table S8.** Time limits. **Figure S1.** EIC m/z 173.0092. **Figure S2.** Isocitrate source fragmentation – MS spectrum. **Figure S3.** cis-Aconitic acid - C_6_H_6_O_6_. **Figure S4.** Isocitric acid – C_6_H_8_O_7_. **Figure S5.** S-Plot - Roots - ESI+. **Figure S6.** S-Plot - Leaves - ESI+. **Figure S7.** S-Plot - Roots - ESI-. **Figure S8.** S-Plot - Leaves - ESI-. **Figure S9.** GSSG relative abundance – Roots. **Figure S10.** GSSG relative abundance – Leaves. **Figure S11.** In silico fragmentations from MS^E^ acquisition of GSSG in roots and leaves. **Figure S12.** Citrate relative abundance – Roots. **Figure S13.** Aspartate relative abundance – Roots. **Figure S14.** Asparagine relative abundance – Roots. **Figure S15.** Extracted ion chromatograms.

## Abbreviations

GC–MS: gas chromatography–mass spectrometry
LC–MS: liquid chromatography–mass spectrometry
HILIC: hydrophilic interaction liquid chromatography
HPLC–MS: high performance liquid chromatography–mass spectrometry
EI: electronic impact
UPLC–HRMS: ultra-high performance liquid chromatography–high resolution mass spectrometry
LC–HRMS: liquid chromatography–high resolution mass spectrometry
QToF MS: quadrupole/time-of-flight mass spectrometer
ESI: electrospray ionization
FWHM: full width half maximum
LOD: limit of detection
LOQ: limit of quantitation
RPM: rotation per minute
QC: quality control
PCA: principal components analysis
OPLS-DA: orthogonal projections to latent structures discriminant analysis
ANOVA: analysis of variance
S-Plots: score plots
RT: retention time
GSH: glutathione reduced
GSSG: glutathione oxidized
SAM: S-adenosyl-methionine
