# Supplementary material for: Simultaneous untargeted and targeted metabolomics profiling of underivatized primary metabolites in sulfur-deficient barley by ultra-high performance liquid chromatography-quadrupole/time-of-flight mass spectrometry
Source: Plant Methods. 2018 Jul 24;14:62. doi: 10.1186/s13007-018-0329-0 (PMC6056915; doi:10.1186/s13007-018-0329-0)
Supplement: Supplementary file 1 — Additional file 1. Table S1. RSD – ESI+ – Lowest concentration. Table S2. RSD – ESI+ – Intermediate concentration. Table S3. RSD – ESI+ – Highest concentration. Table S4. RSD – ESI- – Lowest concentration. Table S5. RSD – ESI- – Intermediate concentration. Table S6. RSD – ESI- – Highest concentration. Table S7. Adducts list. Table S8. Time limits. Figure S1. EIC m/z 173.0092. Figure S2. Isocitrate source fragmentation – MS spectrum. Figure S3. cis-Aconitic acid - C6H6O6. Figure S4. Isocitric acid – C6H8O7. Figure S5. S-Plot - Roots - ESI+. Figure S6. S-Plot - Leaves - ESI+. Figure S7. S-Plot - Roots - ESI-. Figure S8. S-Plot - Leaves - ESI-. Figure S9. GSSG relative abundance – Roots. Figure S10. GSSG relative abundance – Leaves. Figure S11. In silico fragmentations from MSE acquisition of GSSG in roots and leaves. Figure S12. Citrate relative abundance – Roots. Figure S13. Aspartate relative abundance – Roots. Figure S14. Asparagine relative abundance – Roots. Figure S15. Extracted ion chromatograms. [file 13007_2018_329_MOESM1_ESM.docx]

**Supporting Information**

**Simultaneous untargeted and targeted metabolomics profiling of underivatized primary metabolites in sulfur-deficient barley by Ultra-High Performance Liquid Chromatography-Quadrupole/Time-of-Flight Mass Spectrometry**

Hikmat Ghosson^1,2^, Adrián Schwarzenberg^1,*^, Frank Jamois^1^, Jean-Claude Yvin^1^

*^1^Centre Mondial de l’Innovation Roullier (CMI), 18 Avenue Franklin Roosevelt, 35400, Saint-Malo, France.*

*^2^UR1, UFR Sciences et propriétés de la matière, Université de Rennes 1, 2 rue du Thabor, CS 46510, 35065 Rennes Cedex, France*

*Table S1: RSD – ESI+ – Lowest concentration
nc: not calculated*

| **Compound** | **Concentration (µg/L)** | **RSD (%) of RT** | **RSD (%) of PA** |
| --- | --- | --- | --- |
| Proline | 2.5 | 0.70 | 4.81 |
| Isoleucine | 2.5 | 0.56 | 3.60 |
| Leucine | 2.5 | 0.41 | 5.64 |
| Asparagine | 2 | nc | nc |
| Glutamine | 2 | 0.51 | 2.21 |
| Lysine | 3 | 0.00 | 2.57 |
| O-Acetyl-Serine | 2 | nc | nc |
| Methionine | 3 | 0.71 | 4.39 |
| Histidine | 3 | 0.35 | 1.18 |
| Phenylalanine | 4 | 1.71 | 3.35 |
| Arginine | 4 | 0.18 | 7.12 |
| Tyrosine | 4 | 0.00 | 1.48 |
| Tryptophan | 2 | 0.52 | 3.26 |
| Thiamine | 2 | 0.12 | 3.38 |
| Glutathione reduced | 2 | 1.04 | 1.05 |
| S-Adenosyl-Methionine | 2 | 0.51 | 2.49 |
| Glutathione oxidized | 2 | 0.20 | 6.82 |

Table S2: RSD – ESI+ – Intermediate concentration

| **Compound** | **Concentration (µg/L)** | **RSD (%) of RT** | **RSD (%) of PA** |
| --- | --- | --- | --- |
| Proline | 12.5 | 0.68 | 4.98 |
| Isoleucine | 12.5 | 0.29 | 1.78 |
| Leucine | 12.5 | 0.36 | 1.55 |
| Asparagine | 10 | 0.53 | 1.79 |
| Glutamine | 10 | 0.83 | 4.64 |
| Lysine | 15 | 0.00 | 0.76 |
| O-Acetyl-Serine | 10 | 3.00 | 4.51 |
| Methionine | 15 | 0.30 | 3.42 |
| Histidine | 15 | 0.81 | 1.02 |
| Phenylalanine | 20 | 0.00 | 2.04 |
| Arginine | 20 | 0.53 | 2.16 |
| Tyrosine | 20 | 0.32 | 0.41 |
| Tryptophan | 10 | 0.00 | 1.67 |
| Thiamine | 10 | 0.85 | 1.71 |
| Glutathione reduced | 10 | 0.25 | 1.95 |
| S-Adenosyl-Methionine | 10 | 0.86 | 1.59 |
| Glutathione oxidized | 10 | 0.15 | 1.36 |

*Table S3: RSD – ESI+ – Highest concentration*

| **Compound** | **Concentration (µg/L)** | **RSD (%) of RT** | **RSD (%) of PA** |
| --- | --- | --- | --- |
| Proline | 62.5 | 0.75 | 3.81 |
| Isoleucine | 62.5 | 0.40 | 3.30 |
| Leucine | 62.5 | 0.27 | 1.67 |
| Asparagine | 50 | 0.87 | 1.35 |
| Glutamine | 50 | 0.67 | 3.99 |
| Lysine | 75 | 0.00 | 2.01 |
| O-Acetyl-Serine | 50 | 0.57 | 4.86 |
| Methionine | 75 | 0.60 | 6.54 |
| Histidine | 75 | 1.34 | 0.89 |
| Phenylalanine | 100 | 0.24 | 2.36 |
| Arginine | 100 | 0.81 | 1.73 |
| Tyrosine | 100 | 0.99 | 0.22 |
| Tryptophan | 50 | 0.16 | 3.29 |
| Thiamine | 50 | 1.06 | 1.59 |
| Glutathione reduced | 50 | 0.79 | 0.97 |
| S-Adenosyl-Methionine | 50 | 0.43 | 1.03 |
| Glutathione oxidized | 50 | 0.14 | 3.02 |

Table S4: RSD – ESI- – Lowest concentration
nc: not calculated

| **Compound** | **Concentration (µg/L)** | **RSD (%) of RT** | **RSD (%) of PA** |
| --- | --- | --- | --- |
| Fumaric acid | 200 | 0.42 | 1.09 |
| Succinic acid | 200 | 0.37 | 0.96 |
| Aspartic acid | 100 | 0.65 | 0.33 |
| Malic acid | 100 | 0.00 | 0.30 |
| Glutamic acid | 100 | 0.00 | 0.39 |
| Phospho(enol)pyruvic acid | 200 | nc | nc |
| Gallic acid | 100 | 0.14 | 2.62 |
| Cis-Aconitic acid | 100 | 0.43 | 7.72 |
| Shikimic acid | 100 | 0.36 | 1.31 |
| Azelaic acid | 20 | 0 | 1.93 |
| Citric acid | 20 | 0.29 | 4.86 |
| Isocitric acid | 20 | 0.37 | 4.77 |
| Gluconic acid | 20 | 0.48 | 5.30 |
| Kaempferol | 20 | 0.00 | 5.81 |
| Chlorogenic acid | 20 | 0.00 | 3.61 |
| Trehalose 6-Phosphate | 20 | 0.00 | 3.39 |

*Table S5: RSD – ESI- – Intermediate concentration*

| **Compound** | **Concentration (µg/L)** | **RSD (%) of RT** | **RSD (%) of PA** |
| --- | --- | --- | --- |
| Fumaric acid | 1000 | 0.43 | 0.36 |
| Succinic acid | 1000 | 0.38 | 0.33 |
| Aspartic acid | 500 | 0.00 | 0.10 |
| Malic acid | 500 | 0.28 | 0.23 |
| Glutamic acid | 500 | 0.00 | 0.18 |
| Phospho(enol)pyruvic acid | 1000 | 0.00 | 0.43 |
| Gallic acid | 500 | 0.14 | 0.93 |
| Cis-Aconitic acid | 500 | 0.22 | 7.07 |
| Shikimic acid | 500 | 0.41 | 1.83 |
| Azelaic acid | 100 | 0.00 | 0.50 |
| Citric acid | 100 | 0.29 | 2.33 |
| Isocitric acid | 100 | 0.42 | 3.60 |
| Gluconic acid | 100 | 0.48 | 2.17 |
| Kaempferol | 100 | 0.06 | 5.29 |
| Chlorogenic acid | 100 | 0.06 | 1.13 |
| Trehalose 6-Phosphate | 100 | 0.00 | 2.14 |

Table S6: RSD – ESI- – Highest concentration

| **Compound** | **Concentration (µg/L)** | **RSD (%) of RT** | **RSD (%) of PA** |
| --- | --- | --- | --- |
| Fumaric acid | 5000 | 0.00 | 0.15 |
| Succinic acid | 5000 | 0.34 | 0.16 |
| Aspartic acid | 2500 | 0.53 | 0.43 |
| Malic acid | 2500 | 0.00 | 0.66 |
| Glutamic acid | 2500 | 0.00 | 1.25 |
| Phospho(enol)pyruvic acid | 5000 | 0.34 | 2.58 |
| Gallic acid | 2500 | 0.12 | 0.68 |
| Cis-Aconitic acid | 2500 | 0.25 | 3.49 |
| Shikimic acid | 2500 | 0.27 | 0.76 |
| Azelaic acid | 500 | 0.05 | 0.45 |
| Citric acid | 500 | 0.29 | 1.15 |
| Isocitric acid | 500 | 0.00 | 0.81 |
| Gluconic acid | 500 | 0.00 | 0.50 |
| Kaempferol | 500 | 0.08 | 4.69 |
| Chlorogenic acid | 500 | 0.00 | 0.96 |
| Trehalose 6-Phosphate | 500 | 0.31 | 0.88 |

*Table S7: Adducts list*

| **Adduct** | **Adduct mass** | **Charge** |
| --- | --- | --- |
| *ESI+* |  |  |
|  |  |  |
| M+3H | 3.0218 | 3 |
| M+2H+Na | 25.0038 | 3 |
| M+2Na+H | 46.9857 | 3 |
| M+2H | 2.0146 | 2 |
| M+H+Na | 23.9965 | 2 |
| M+2Na | 45.9784 | 2 |
| M+H-2H_2_O | -35.0139 | 1 |
| M+H-H_2_O | -17.003 | 1 |
| M+H | 1.0073 | 1 |
| 2M+3H_2_O+2H | 28.0231 | 2 |
| M+NH_4_ | 18.0338 | 1 |
| M+Na | 22.9892 | 1 |
| M+CH_3_OH+H | 33.0335 | 1 |
| M+K | 38.9632 | 1 |
| M+ACN+H | 42.0338 | 1 |
| M+2Na-H | 44.9712 | 1 |
| M+ACN+Na | 64.0158 | 1 |
| 2M+H | 1.0073 | 1 |
| 2M+NH_4_ | 18.0338 | 1 |
| 2M+Na | 22.9892 | 1 |
| 2M+K | 38.9632 | 1 |
| 2M+ACN+H | 42.0338 | 1 |
| 2M+ACN+Na | 64.0158 | 1 |
|  |  |  |
| *ESI-* |  |  |
|  |  |  |
| M-3H | -3.0218 | -3 |
| M-2H | -2.0146 | -2 |
| M-H_2_O-H | -19.0178 | -1 |
| M-H | -1.0073 | -1 |
| M+Na-2H | 20.9747 | -1 |
| M+Cl | 34.9694 | -1 |
| M+K-2H | 36.9486 | -1 |
| M+FA-H | 44.9982 | -1 |
| 2M-H | -1.0073 | -1 |
| 2M+FA-H | 44.9982 | -1 |
| 2M+Hac-H | 59.0139 | -1 |
| 3M-H | -1.0073 | -1 |

Table S8: Time limits

| Ionization polarity | Sample type | Time limits (min) |
| --- | --- | --- |
| ESI+ | Leaves | 0.4-5.25 |
| ESI+ | Roots | 0.45-4 |
| ESI- | Leaves | 0.65-9.5 |
| ESI- | Roots | 0.77-9.75 |


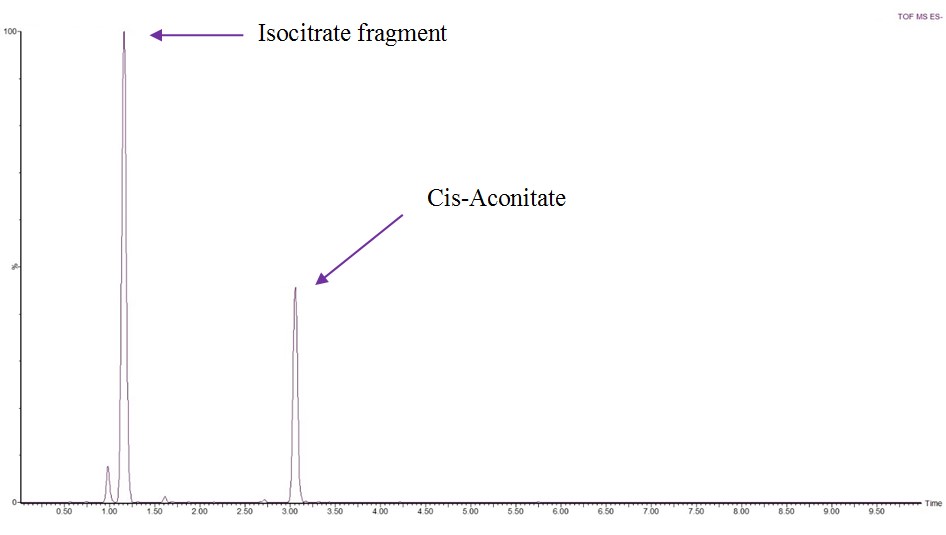


Figure S1: EIC m/z 173.0092


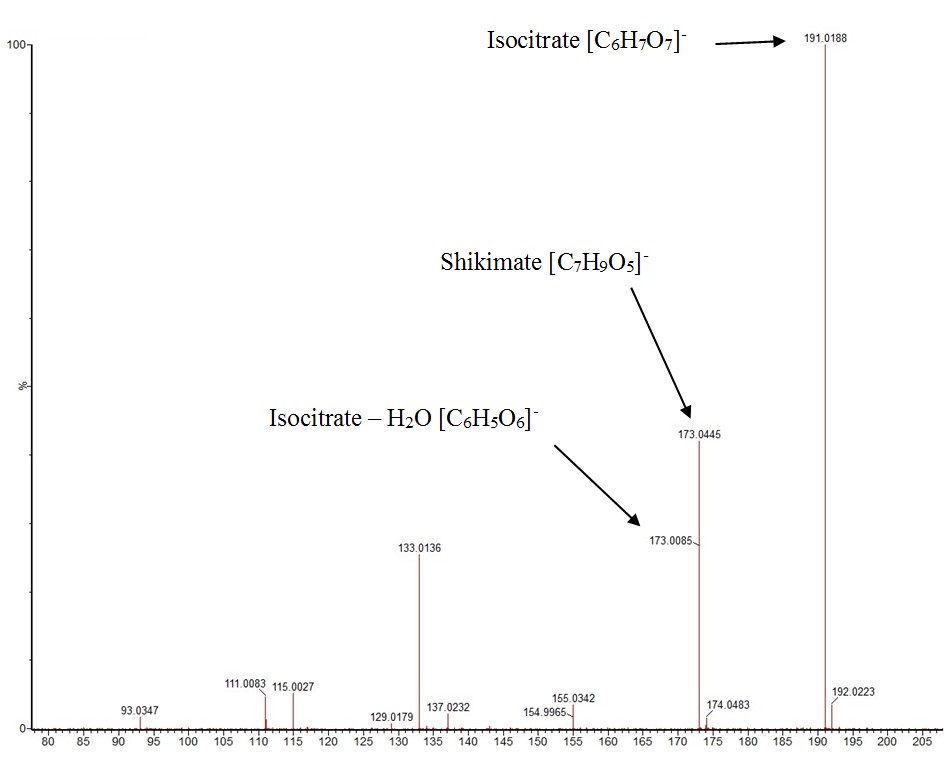


Figure S2: Isocitrate source fragmentation – MS spectrum

| 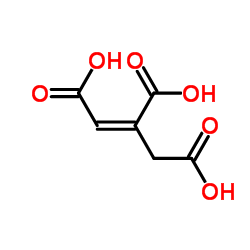  Figure S3: cis-Aconitic acid - C_6_H_6_O_6_ | 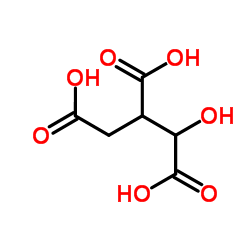  Figure S4: Isocitric acid – C_6_H_8_O_7_ |
| --- | --- |


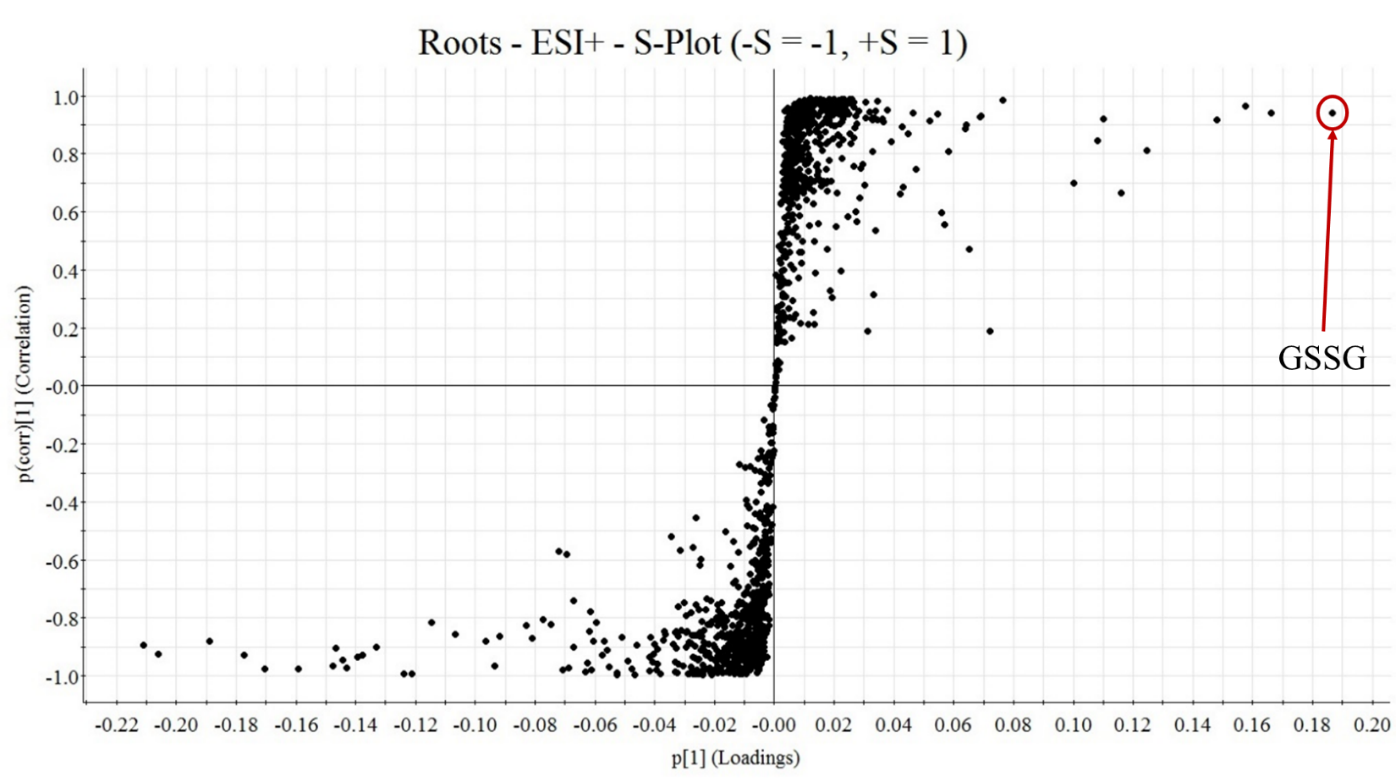


Figure S5: S-Plot - Roots - ESI+


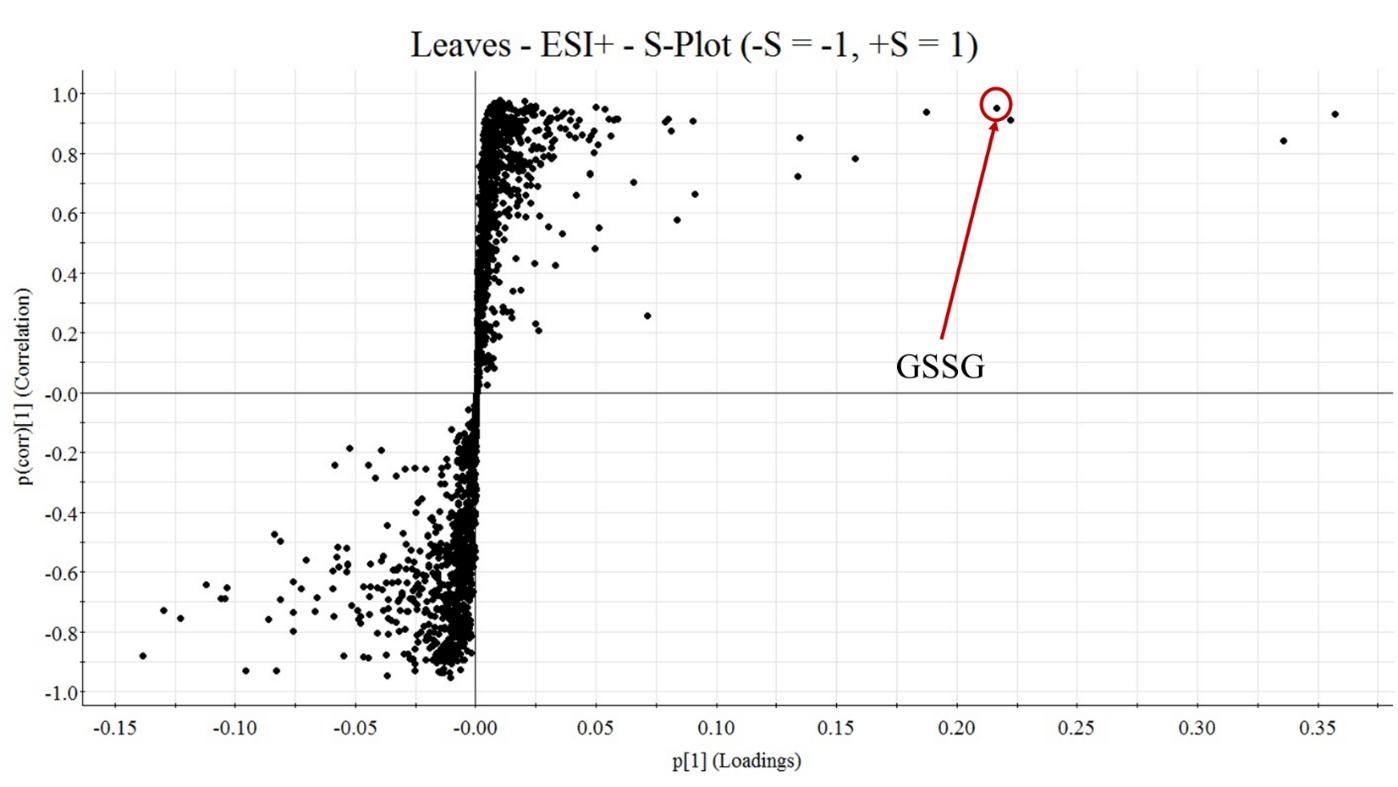


Figure S6: S-Plot - Leaves - ESI+


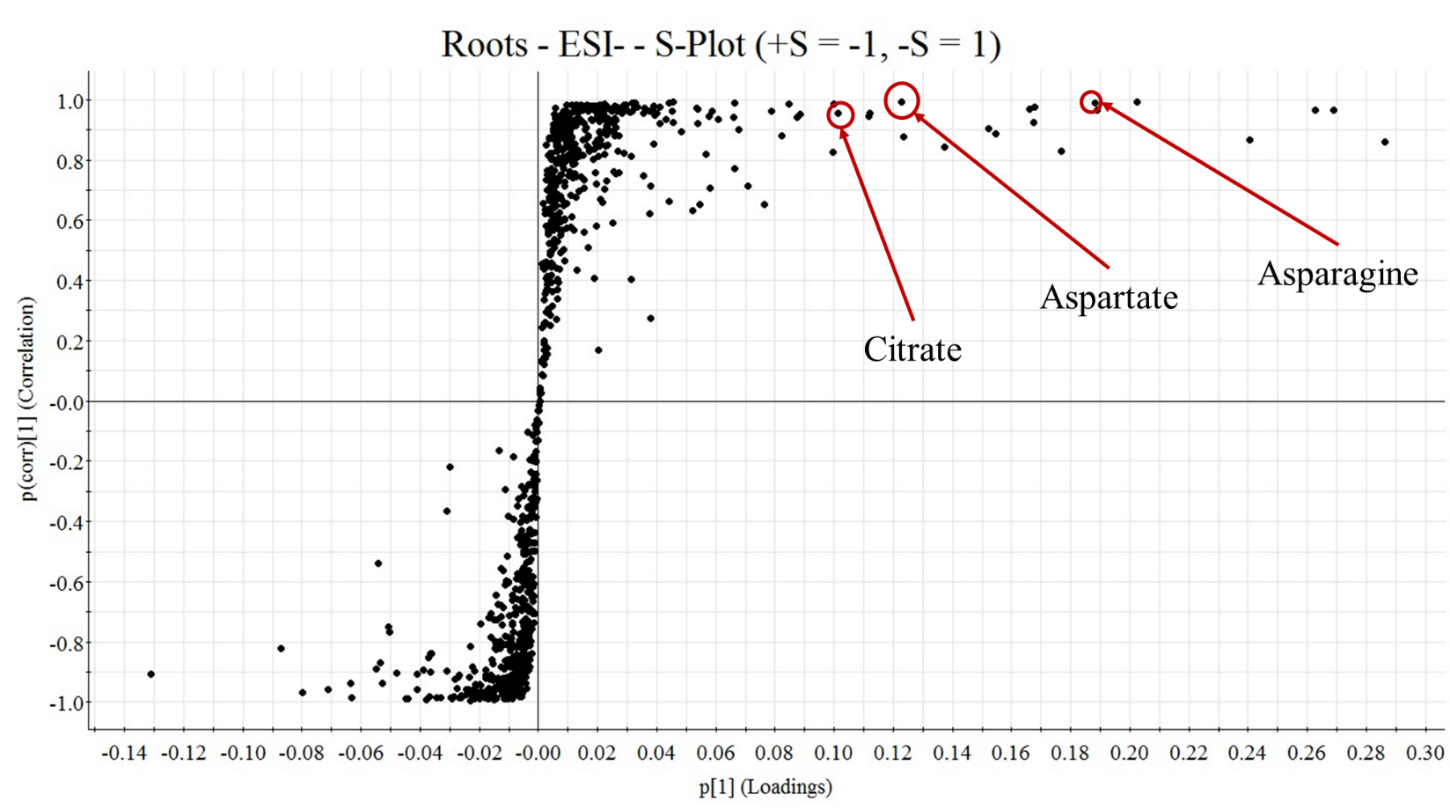


Figure S7: S-Plot - Roots - ESI-


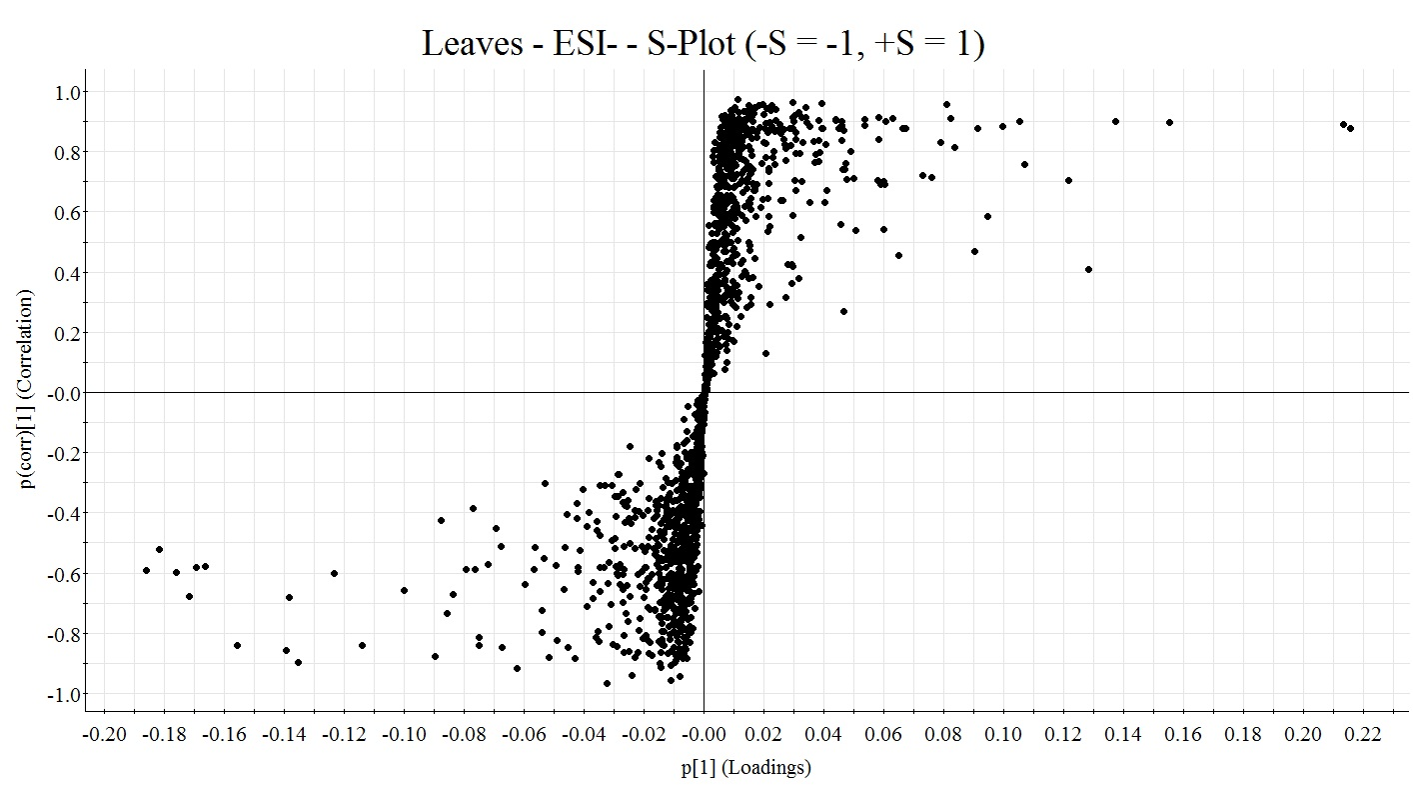


Figure S8: S-Plot - Leaves - ESI-


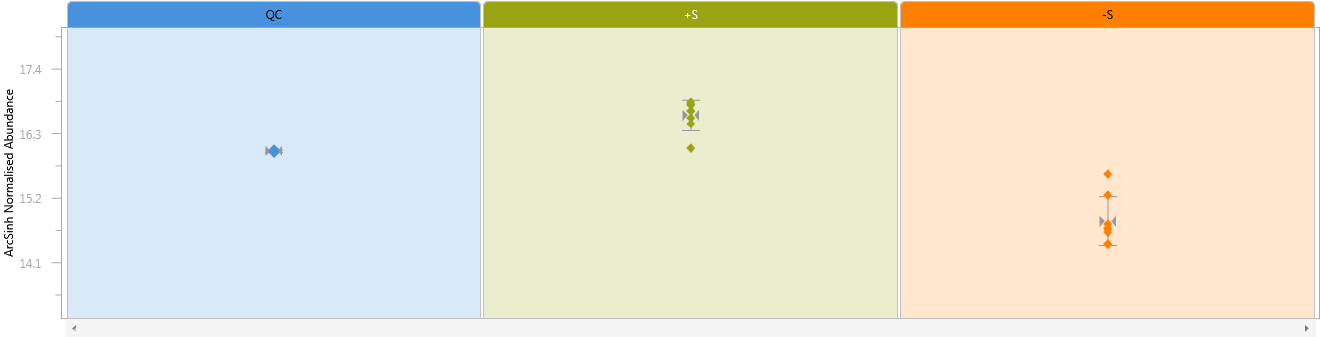


Figure S9: GSSG relative abundance – Roots


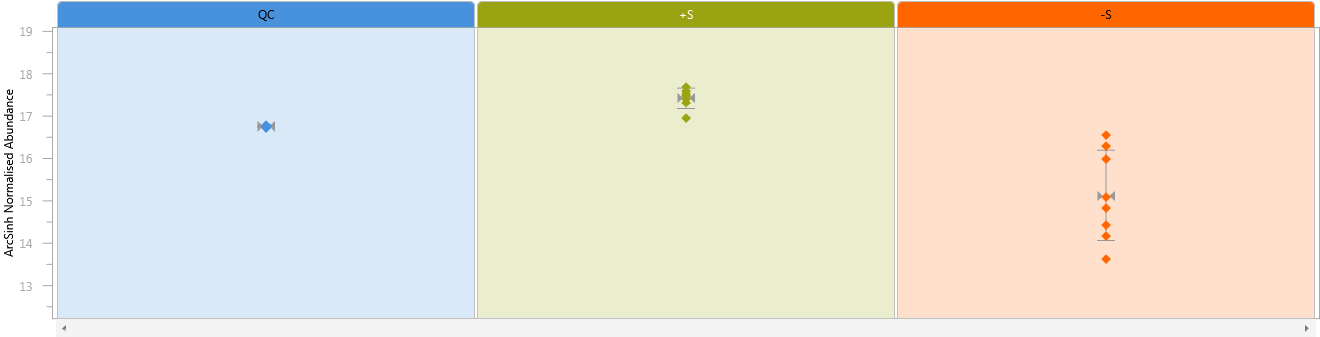


Figure S10: GSSG relative abundance – Leaves


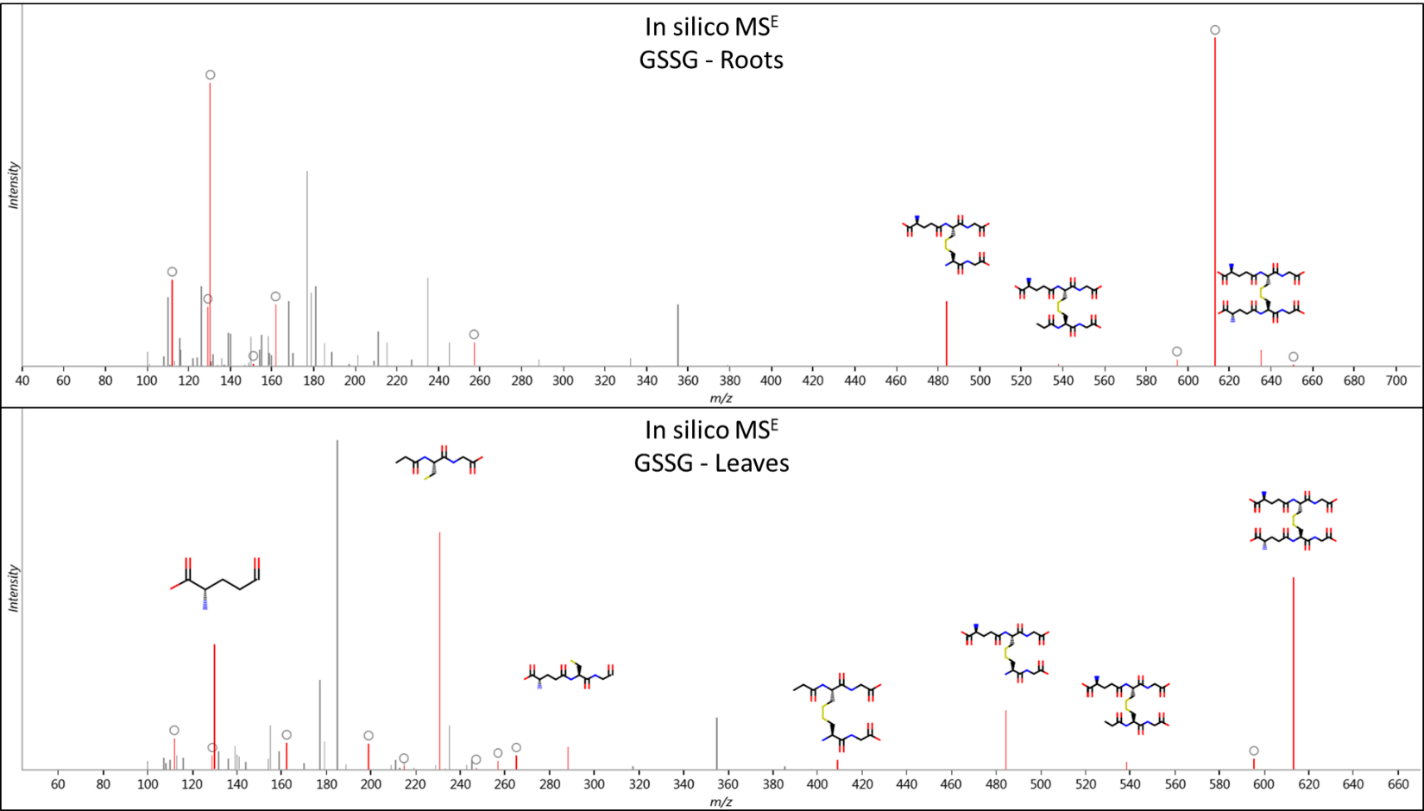


Figure S11: In silico fragmentations from MS^E^ acquisition of GSSG in roots and leaves


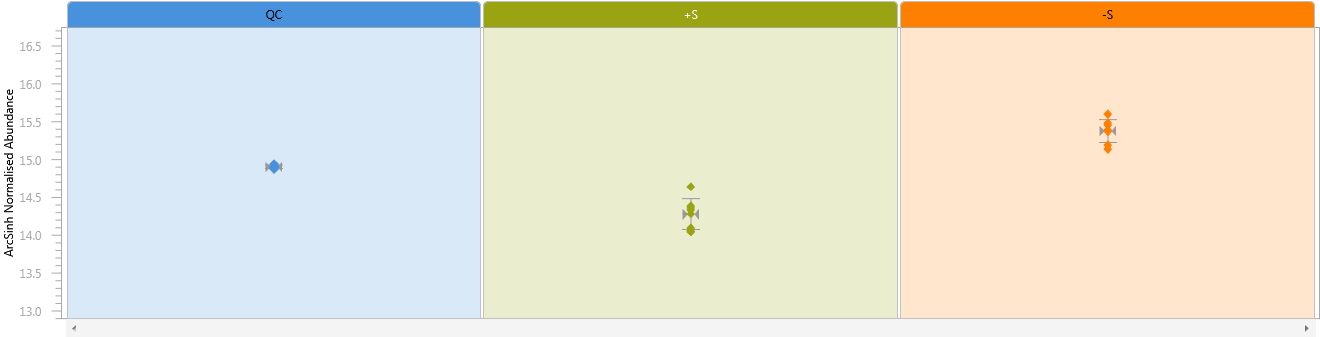


Figure S12: Citrate relative abundance – Roots


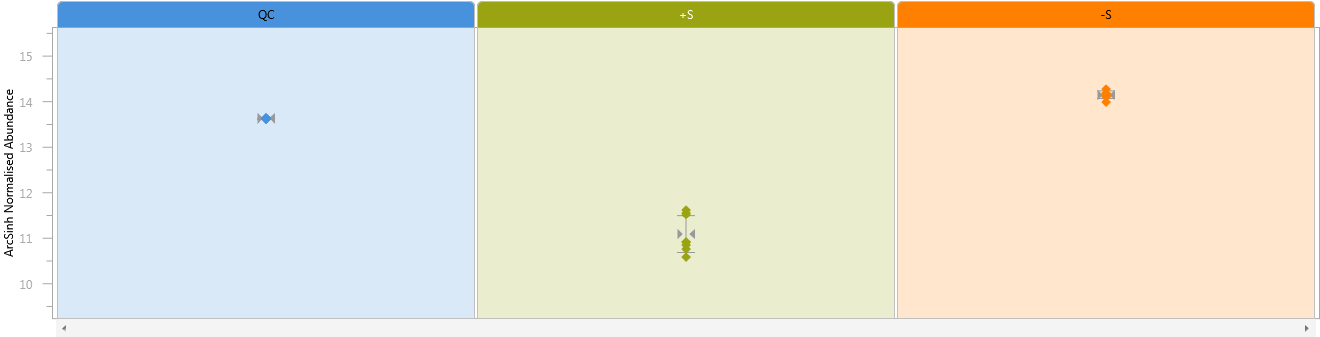


Figure S13: Aspartate relative abundance – Roots


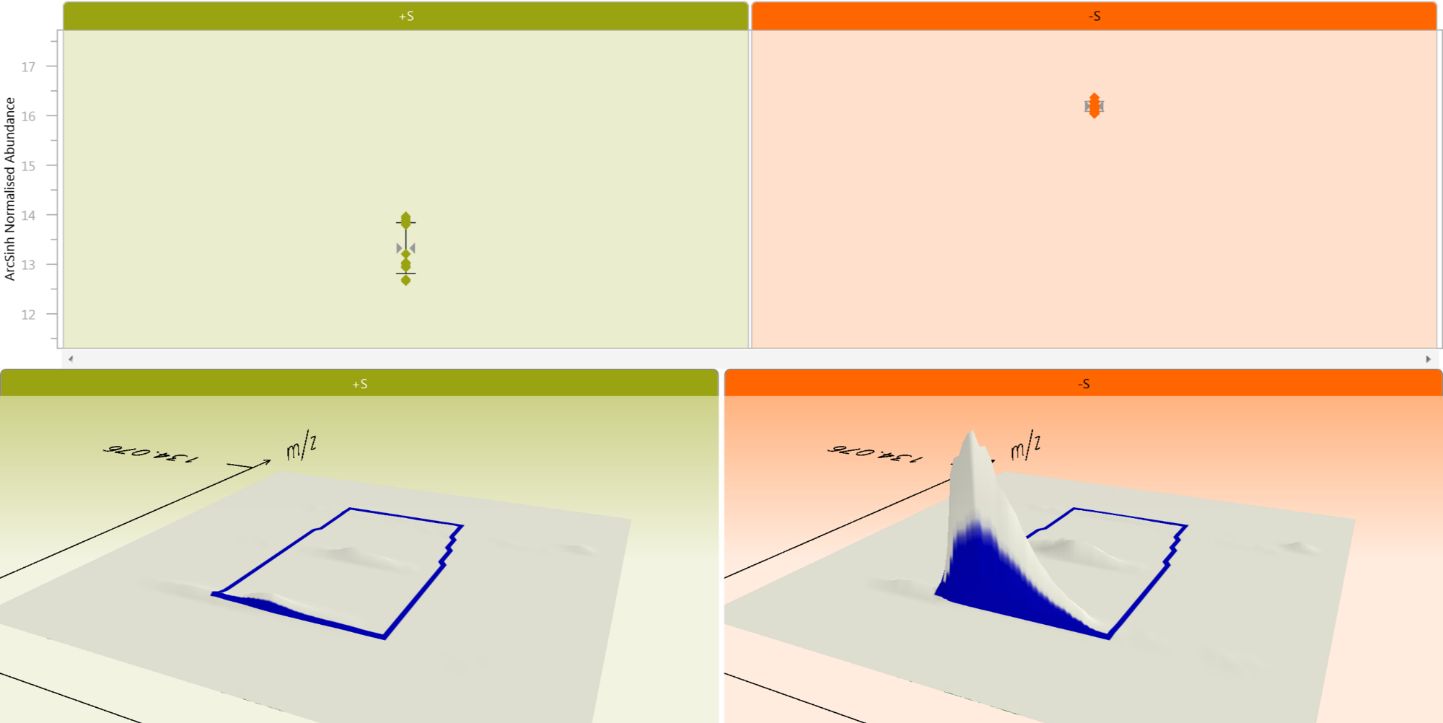


Figure S14: Asparagine relative abundance – Roots


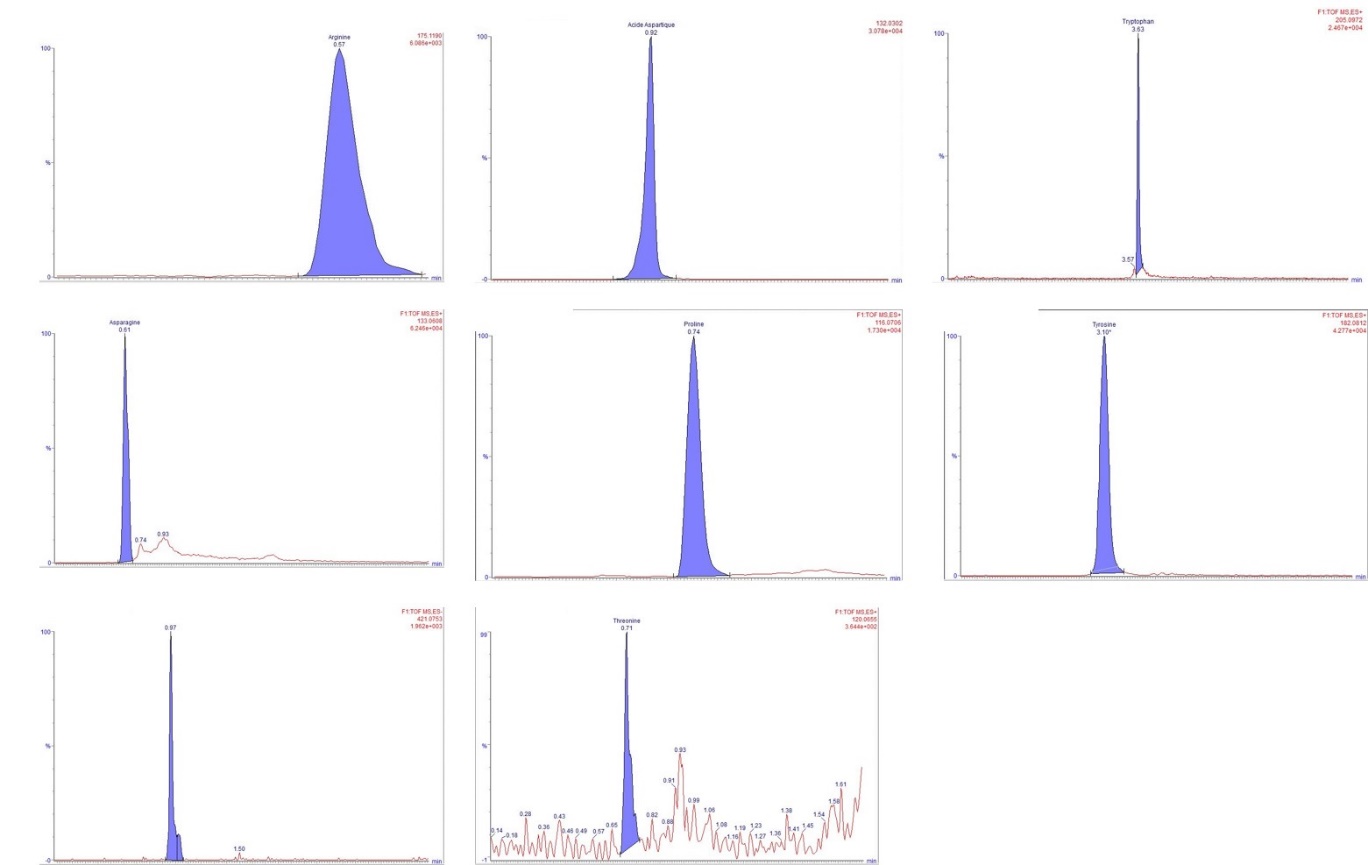

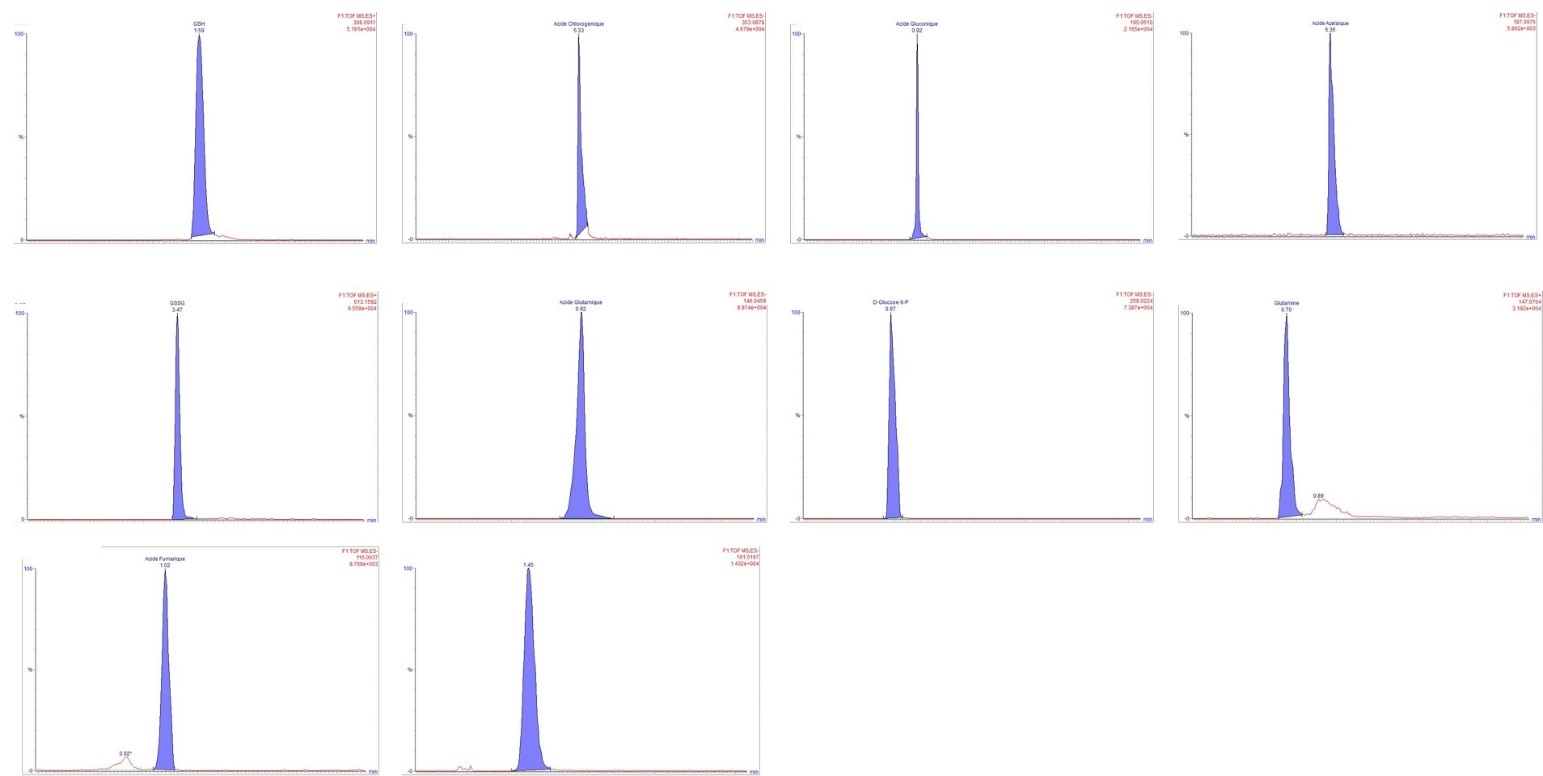

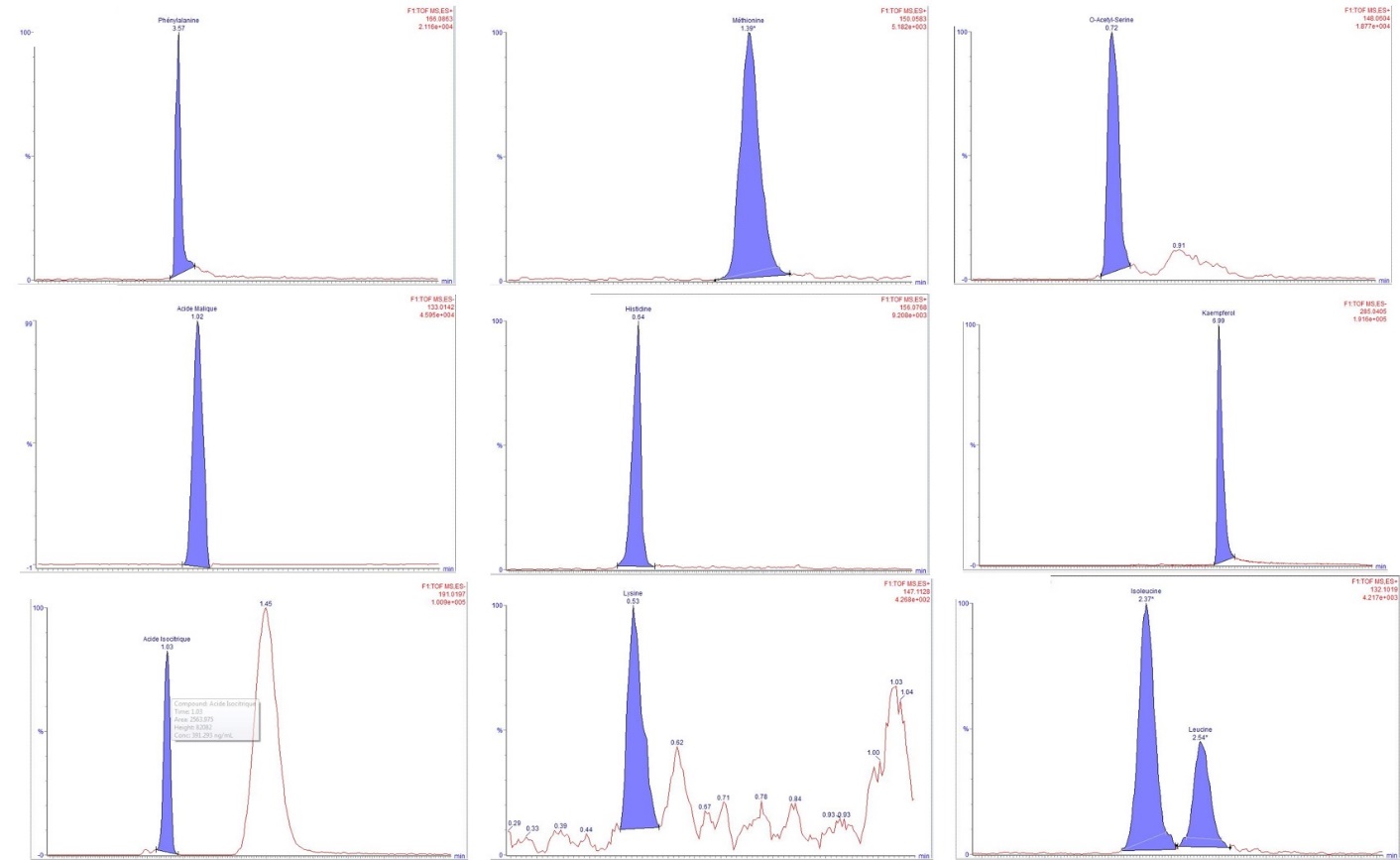


Figure S15: Extracted ion chromatograms
